# Infant Growth After Mass Administration of Azithromycin: Secondary Outcomes of a Cluster Randomized Clinical Trial

**DOI:** 10.1001/jamanetworkopen.2026.17425

**Published:** 2026-06-10

**Authors:** Laura Adubra, Juho Luoma, Yue-Mei Fan, Fadima Cheick Haidara, Oumar Samaké, Rikhard Ihamuotila, Kaisa Ylikruuvi, Lotta Hallamaa, Owen Martell, Camilla Ducker, Dagmar Alber, Per Ashorn, Yin Bun Cheung, Samba Sow, Ulla Ashorn

**Affiliations:** 1 Center for Child, Adolescent and Maternal Health Research, Faculty of Medicine and Health Technology, Tampere University, Tampere, Finland; 2 Center for Vaccine Development–Mali, Bamako, Mali; 3 Tro Da Ltd, Caerphilly, Wales, United Kingdom; 4 Great Ormond Street Institute of Child Health, University College London, London, United Kingdom

## Abstract

**Importance:**

Mass administration of azithromycin reduced child mortality in parts of sub-Saharan Africa. Promotion of infant growth may be an additional benefit of azithromycin treatment.

**Objective:**

To investigate whether the provision of mass administration of azithromycin for infants aged 1 to 11 months improves growth outcomes.

**Design, Setting, and Participants:**

This cluster randomized clinical trial conducted from December 1, 2020, to December 31, 2024, included 1151 villages in southwestern Mali. Villages were randomized in a 3:4:2 ratio to placebo, twice-yearly azithromycin, or quarterly azithromycin. Participants and study personnel were masked. The prespecified growth substudy, conducted from August 31, 2022, to July 31, 2023, was conducted in 59 villages in the Kita region. Eligible participants were infants aged 1 to 11 months, weighing 3.0 kg or more, with no macrolide allergy or severe illness. Anthropometric data were collected cross-sectionally from children aged 6 to 8 months and 12 to 14 months at 15, 18, 21, and 24 months after village enrollment.

**Interventions:**

Infants received a single oral dose of azithromycin, 20 mg/kg, at each quarterly visit: placebo at all rounds (control); azithromycin at 2 rounds from January to June and placebo at 2 rounds from July to December (twice-yearly azithromycin); or azithromycin at all rounds (quarterly azithromycin).

**Main Outcomes and Measures:**

Prespecified outcomes were weight and length; weight-for-age (WA), length-for-age (LA), weight-for-length (WL), and mid-upper-arm circumference (MUAC) *z* scores; and underweight, stunting, and wasting. Analysis was performed on an intention-to-treat basis.

**Results:**

A total of 1205 infants (mean age, 5.7 months [95% CI, 5.6-5.9 months]; 633 boys [52.5%]) were included in the trial at baseline. Among 1789 children (923 boys [51.6%]) assessed for growth, the mean anthropometric indices did not differ between the placebo, twice-yearly azithromycin, and quarterly azithromycin groups: weight, 7.8 kg (95% CI, 7.7-7.9 kg), 7.8 kg (95% CI, 7.7-7.9 kg), and 7.8 kg (95% CI, 7.7-7.9 kg), respectively; length, 70.2 cm (95% CI, 69.9-70.8 cm), 70.6 cm (95% CI, 70.2-70.9 cm), and 70.6 cm (95% CI, 70.1-71.0 cm), respectively; WA *z* score, –1.12 (95% CI, −1.23 to −0.95), −1.10 (95% CI, −1.19 to −0.98), and −1.12 (95% CI, −1.27 to −1.01), respectively; LA *z* score, −0.86 (95% CI, −1.06 to −0.70), –0.79 (95% CI, −0.93 to −0.65), and –0.78 (95% CI, −0.98 to −0.63), respectively; WL *z* score, –0.87 (95% CI, −1.00 to −0.68), −0.89 (95% CI, −1.01 to −0.77), and −0.95 (95% CI, −1.12 to −0.81), respectively; and MUAC *z* score, −0.30 (95% CI, −0.43 to −0.10), −0.24 (95% CI, −0.37 to −0.12), and −0.26 (95% CI, −0.41 to −0.10), respectively. The proportions of underweight, stunting, and wasting did not differ between groups.

**Conclusions and Relevance:**

In this prespecified analysis of secondary outcomes of a cluster randomized clinical trial of the provision of mass administration of azithromycin for infants aged 1 to 11 months, growth outcomes did not differ between azithromycin and placebo groups. These findings suggest that mass administration of azithromycin to infants is unlikely to promote growth.

**Trial Registration:**

ClinicalTrials.gov Identifier: NCT04424511

## Key Points

**Question:**

Does mass administration of azithromycin promote infant growth?

**Findings:**

This analysis of secondary outcomes of a 3-arm cluster randomized clinical trial in Mali in which all infants aged 1 to 11 months received placebo, azithromycin twice yearly, or azithromycin quarterly evaluated infant growth at 15, 18, 21, and 24 months after village enrollment. Anthropometric assessments among 1789 children aged 6 to 8 months and 12 to 14 months showed no significant differences between groups in mean weight and length; weight-for-age, length-for-age, weight-for-length, or mid-upper-arm circumference *z* scores; or the prevalence of underweight, stunting, or wasting.

**Meaning:**

These findings do not support a hypothesis that mass administration of azithromycin promotes infant growth.

## Teaser

This analysis of secondary outcomes of a randomized clinical trial investigates whether the provision of mass administration of azithromycin for infants aged 1 to 11 months improves growth outcomes.

## Introduction

Mass drug administration (MDA) of azithromycin has been associated with reduced child mortality in sub-Saharan Africa.^1,2,3,4,5^ Growth promotion may be an additional benefit of azithromycin treatment, potentially mediated through mechanisms that overlap with those hypothesized to improve survival, including reduced infection and inflammation and improved gut health.^6^ Exposure to antibiotics, particularly macrolides, during the first 6 months of life has been associated with increased weight up to 2 years of age in low-resource settings^7^ and in a Finnish cohort.^8^ Investigating azithromycin’s effects on infant growth is especially relevant considering the 2023 World Health Organization (WHO) guideline on the prevention and management of wasting and nutritional edema, which extended the scope of earlier guidance to a broader population of concern—infants younger than 6 months at risk of poor growth and development—and identified the evaluation of routine antibiotics in this group as a research priority.^9,10^ To date, trials of azithromycin MDA for trachoma control or child survival have not demonstrated overall growth benefits, although possible effects have been suggested among the most vulnerable.^11,12,13,14,15,16,17,18^

From December 1, 2020, through December 1, 2024, the Large-Scale Assessment of the Key Health-Promoting Activities of Two New Mass Drug Administration Regimens With Azithromycin (LAKANA) trial was conducted in Mali to evaluate the effect on mortality of azithromycin MDA to infants aged 1 to 11 months.^19^ A previous study reported no reduction in infant or child mortality.^20^ In this analysis, we present findings on prespecified secondary growth outcomes, including weight and length; weight-for-age (WA), length-for-age (LA), weight-for-length (WL), and mid-upper-arm circumference (MUAC) *z* scores; and underweight, stunting, and wasting.

## Methods

### Study Design

LAKANA was a large-scale, cluster randomized, double-blinded, 3-arm clinical trial designed to assess the effect of administering 4 or 2 annual rounds of azithromycin MDA to infants aged 1 to 11 months on infant and child mortality in Mali (trial protocol in Supplement 1).^19^ Villages (clusters) were randomized in a 3:4:2 ratio to placebo, twice-yearly azithromycin, and quarterly azithromycin, respectively; this allocation was selected to ensure adequate statistical power for the primary mortality comparisons in the parent trial. Nine trial visits occurred at 3-month intervals: 8 MDA rounds in which infants were treated and 1 final close-out visit without drug administration (eFigure 1 in Supplement 2). Seasonal malaria chemoprevention, from July through December, was the sole cointervention in all communities and was provided by the national program. The trial protocol and statistical analysis plan have been published,^19,21^ and the full protocol is available at the trial website.^22^ Ethics approval was obtained from the Mali institutional review board, the Comité d’Éthique de l’Université des Sciences, des Techniques et des Technologies de Bamako, and from the ethics committee of the Pirkanmaa Hospital District. Study staff obtained village leaders’ oral permission and informed oral consent confirmed with a digital signature from household heads or proxies for trial and growth study participation. This study is reported following the Consolidated Standards of Reporting Trials (CONSORT) reporting guideline for cluster randomized clinical trials.

The growth substudy, conducted from August 31, 2022, to July 31, 2023, was conducted in 59 villages in Kita region, selected on proximity to health centers for logistical reasons, and forming the trial’s prespecified secondary outcome sample. It used a repeated cross-sectional design with anthropometric assessments at 15, 18, 21, and 24 months after village enrollment, corresponding to MDA rounds 6, 7, and 8, and the close-out visit (eFigure 1 in Supplement 2). Different cohorts of children aged 6 to 8 months and 12 to 14 months were examined at each visit. This sampling frame was chosen to ensure maximal exposure to the study drug at size assessment; children aged 6 to 8 months would have received up to 2 doses of azithromycin, and those aged 12 to 14 months would have received up to 4 doses.

### Trial Eligibility

All infants aged 1 to 11 months, weighing at least 3.0 kg, and with no macrolide allergy or severe illness, were eligible to receive study treatment. At baseline (MDA 1) and at each subsequent visit, study teams conducted and reviewed a household census to enumerate all residents and identify infants. Age was calculated by the data collection software as the difference between the visit date and the date of birth recorded on the child’s health card (or other identification document when the health card was unavailable). The composition of the eligible population varied across visits as infants were born, absent, or aged out of eligibility.

### Randomization and Masking

Randomization was stratified by cluster size (small, <100 infants; large, ≥100 infants), based on national population estimates. Research Triangle Institute International, the data support partner at trial initiation, randomly assigned 18 letters of the alphabet to azithromycin or placebo, used in 2-letter combinations to allocate villages to control, twice-yearly treatment, or quarterly treatment. Pfizer Inc donated the study drugs, which were identical in appearance, taste, smell, and packaging except for the treatment letter on the label.

Center for Vaccine Development Mali coordinated village enrollment and organized public allocation events. Study arm allocation was masked to participants, site staff, and investigators. Two of us (L.A. and J.L.) were unblinded at the time of code opening for the primary outcome (mortality) analysis.

### Eligibility for Monitoring Visits

In 59 villages, children aged 6 to 8 months and 12 to 14 months from consenting households were eligible for anthropometric assessments. At each growth substudy visit, field teams visited all households participating in the parent trial and assessed children who met the age-based eligibility criteria at that time.

### Anthropometric Assessment

Anthropometric assessments performed at temporary village-based facilities, with caregivers present, included weight, recumbent length, and MUAC. Weight was measured with an electronic scale (SECA 354; SECA GmbH) with 10-g precision, length with a commercial length board (ShorrBoard; Weigh and Measure LLC) to the nearest 0.1 cm, and MUAC with a nonstretchable tape (Tricolor Shorr Child MUAC tape) to the nearest 1 mm. All anthropometric measures were taken in triplicate. Triplicate measurements had coefficients of variation of less than 1%, indicating minimal variability; their mean values were used in analyses.

Study staff assessed bilateral pitting edema. Caregivers of children with edema or acute malnutrition based on MUAC screening were informed and referred to health facilities.

Devices were inspected daily and calibrated before data collection; failures were replaced. An independent trial monitor visited sites periodically, and refresher training occurred as needed.

Data were collected using a custom-built application (CommCare, Dimagi) and uploaded to a secure cloud server. Integrated logic checks, required fields, and range limits ensured real-time data validation at data entry.

### Intervention

Study staff weighed infants at their homes at each MDA round using an electronic scale (Model M111600-01; ADE Germany GmbH) and administered placebo or azithromycin suspension as a single oral dose of 20 mg/kg, as per WHO 2020 guidelines.^23^ Each infant could receive a total of 1 to 4 doses of the study drug, depending on their age at enrollment. If vomiting occurred within 15 minutes, the dose was repeated. Adverse events were formally assessed by caregiver interview 14 days after MDA among infants aged 4 to 11 months in the 59 secondary outcome villages; these results are reported elsewhere.^24^

### Outcomes

Prespecified anthropometric outcomes included indices reflecting both linear and ponderal growth: weight in kilograms, WA *z* score, and moderate-to-severe underweight (WA *z* score <− 2) or severe underweight (WA *z* score <−3). Linear growth outcomes were length in centimeters, LA *z* score, and moderate-to-severe stunting (LA *z* score <−2) or severe stunting (LA *z* score <−3). Ponderal growth outcomes were MUAC *z* score, WL *z* score, and moderate-to-severe wasting (WL *z* score <−2) or severe wasting (WL *z* score <−3). All outcomes were prespecified secondary end points of the parent mortality trial. Anthropometric *z* scores were derived using the 2006 WHO Child Growth Standards.^25^ Outlier values were defined according to WHO recommendations (WA *z* score <−6 or >5 SD; LA *z* score <−6 or >6 SD; WL *z* score <−5 or >5 SD). Children with outlier values were excluded from all analyses.

We conducted an exploratory analysis among children with repeated measurements (ie, those who remained age eligible and were reassessed at subsequent visits). Weight gain and length gain were calculated as the difference between visits, in grams per day and millimeters per day, respectively.

### Study Population

Children were analyzed following the intention-to-treat principle (ie, according to their village level treatment assignment regardless of the number of doses received). Table 1 presents a baseline snapshot of infants aged 1 to 11 months residing in each village at the time of MDA1 (village enrollment), whereas Table 2 and Table 3 include children aged 6 to 8 months or 12 to 14 months at the time of the growth substudy visits (MDA6-8 and close-out visit).

**Table 1.** Baseline Cluster- and Individual-Level Characteristics at Enrollment^a^

Characteristic	Control	Azithromycin	
		Twice yearly	Quarterly
Cluster level			
No. of villages	20	27	12
Total No. of infants aged 1-11 mo	341	494	370
No. of infants aged 1-11 mo per village, median (IQR)	14 (9-25)	15 (9-27)	14 (7-54)
Distance of village to nearest health center, mean (SD), km	6.2 (2.8)	6.9 (3.6)	6.0 (2.5)
Individual level			
Infant girls, No./total No. (%)	170/341 (49.9)	231/494 (46.8)	171/370 (46.2)
Infant boys, No./total No. (%)	171/341 (50.1)	263/494 (53.2)	199/370 (53.8)
Infant age, overall, mean (95% CI), mo	5.5 (5.2 to 5.9)	5.9 (5.6 to 6.1)	5.6 (5.2 to 6.0)
Infant age, girls, mean (95% CI), mo	5.5 (5.1 to 5.9)	5.8 (5.5 to 6.1)	5.5 (5.1 to 5.9)
Infant age, boys, mean (95% CI), mo	5.6 (5.3 to 6.0)	5.9 (5.6 to 6.3)	5.7 (5.2 to 6.1)
Infant weight, overall, mean (95% CI), kg	6.3 (6.1 to 6.5)	6.5 (6.3 to 6.6)	6.4 (6.1 to 6.6)
Infant weight, girls, mean (95% CI), kg	6.1 (5.9 to 6.3)	6.2 (6.0 to 6.4)	6.1 (5.9 to 6.4)
Infant weight, boys, mean (95% CI), kg	6.6 (6.4 to 6.8)	6.7 (6.5 to 6.9)	6.6 (6.4 to 6.9)
Infant WA *z* score, overall, mean (95% CI)	−1.04 (−1.27 to −0.81)	−1.03 (−1.23 to −0.84)	−0.91 (−1.21 to −0.60)
Infant WA *z* score, girls, mean (95% CI)	−0.94 (−1.18 to −0.70)	−0.93 (−1.14 to −0.72)	−0.81 (−1.12 to −0.49)
Infant WA *z* score, boys, mean (95% CI)	−1.13 (−1.38 to −0.89)	−1.12 (−1.33 to −0.91)	−1.00 (−1.32 to −0.69)
Underweight (WA *z* score <−2) infants overall, No./total No. (%)	84/341 (24.6)	115/494 (23.3)	76/370 (20.5)
Underweight (WA *z* score <−2) infant girls, No./total No. (%)	28/170 (16.5)	49/231 (21.2)	31/171 (18.1)
Underweight (WA *z* score <−2) infant boys, No./total No. (%)	56/171 (32.7)	66/263 (25.1)	45/199 (22.6)
Households at or below the median asset index, No./total No. (%)^b^	113/310 (36.5)	213/464 (45.9)	151/341 (44.3)
Households at or below the median WASH index, No./total No. (%)^b^	159/310 (51.3)	276/464 (59.5)	199/341(58.4)

Abbreviations: WA, weight-for-age; WASH, water, sanitation, and hygiene.

^a^ Infant characteristics are summarized at the individual level for infants aged 1 to 11 months residing in each village at enrollment. Continuous variables are presented as individual-level mean values with 95% CIs adjusted for clustering by village, skewed variables are presented as median (IQR) values, and categorical variables are presented as number (percentage).

^b^ Household asset and WASH indices were derived using principal component analysis of household-level variables among the 59 substudy villages and standardized within this population. Median cutpoints used to define “at or below median” were based on the overall distribution across all substudy villages.

**Table 2.** Anthropometric Outcomes by Treatment Group Among Children Aged 6 to 8 Months and 12 to 14 Months^a^

Outcome	Control (n = 499)	Azithromycin		Difference in mean values (95% CI)		*P* value
		Twice yearly (n = 758)	Quarterly (n = 532)	Twice-yearly azithromycin vs control^b^^,^^c^	Quarterly azithromycin vs control	
Weight, mean (95% CI), kg^b^	7.8 (7.7 to 7.9)	7.8 (7.7 to 7.9)	7.8 (7.7 to 7.9)	0.03 (−0.1 to 0.2)	−0.02 (−0.2 to 0.2)	.77
Length, mean (95% CI), cm	70.2 (69.9 to 70.8)	70.6 (70.2 to 70.9)	70.6 (70.1 to 71.0)	0.2 (−0.3 to 0.8)	0.2 (−0.4 to 0.9)	.54
WA *z* score, mean (95% CI)	−1.12 (−1.23 to −0.95)	−1.10 (−1.19 to −0.98)	−1.12 (−1.27 to −1.01)	0.01 (−0.16 to 0.17)	−0.05 (−0.25 to 0.15)	.74
LA *z* score, mean (95% CI)	−0.86 (−1.06 to −0.70)	−0.79 (−0.93 to −0.65)	−0.78 (−0.98 to −0.63)	0.09 (−0.12 to 0.30)	0.07 (−0.18 to 0.33)	.60
WL *z* score, mean (95% CI)	−0.87 (−1.00 to −0.68)	−0.89 (−1.01 to −0.77)	−0.95 (−1.12 to −0.81)	−0.04 (−0.23 to 0.14)	−0.12 (−0.35 to 0.10)	.43
MUAC *z* score, mean (95% CI)	−0.30 (−0.43 to −0.10)	−0.24 (−0.37 to −0.12)	−0.26 (−0.41 to −0.10)	0.02 (−0.17 to 0.20)	0.01 (−0.22 to 0.23)	.98

Abbreviations: LA, length-for-age; MUAC, mid-upper-arm circumference; WA, weight-for-age; WL, weight-for-length.

^a^ Data in the first 3 columns present individual-level observed mean values based on anthropometric measurements pooled across the 15-, 18-, 21-, and 24-month visits; 95% CIs account for clustering by village. The number per arm reflects the number of unique children who contributed at least 1 measurement; a subset of children contributed repeated measurements at different visits.

^b^ Adjusted estimates were obtained from mixed-effects models accounting for clustering at the village level and within-child correlation via random intercepts and adjusted for village size and child age group.

^c^ Overall comparison across intervention groups was conducted with the likelihood ratio test.

**Table 3.** Underweight, Stunting, and Wasting by Treatment Group and Age Group^a^

Outcome	Control	Azithromycin		Difference in proportions, pp (95% CI)^b^^,^^c^		*P* value
		Twice yearly	Quarterly	Twice-yearly azithromycin vs Control	Quarterly azithromycin vs Control	
**Age group: 6-8 mo**						
No.	300	422	304	NA	NA	NA
Underweight (WA *z* score <−2), No. (%)	50 (16.7)	76 (18.0)	44 (14.5)	1.8 (−3.3 to 6.8)	−0.3 (−6.3 to 5.6)	.67
Severely underweight (WA *z* score <−3), No.(%)	10 (3.3)	19 (4.5)	10 (3.3)	0.9 (−2.2 to 4.1)	−0.3 (−3.9 to 3.3)	.71
Stunting (LA *z* score <−2), No. (%)	26 (8.7)	39 (9.2)	30 (9.9)	0.6 (−3.4 to 4.6)	1.5 (−3.4 to 6.3)	.84
Severe stunting (LA *z* score <−3), No. (%)	4 (1.3)	6 (1.4)	8 (2.6)	0.03 (−1.7 to 1.8)	1.1 (−1.3 to 3.5)	.56
Wasting (WL *z* score <−2), No. (%)	48 (16.0)	73 (17.3)	54 (17.8)	1.1 (−4.6 to 6.8)	2.5 (−4.5 to 9.5)	.77
Severe wasting (WL *z* score <−3), No. (%)	7 (2.3)	13 (3.1)	10 (3.3)	0.4 (−2.6 to 3.3)	−0.1 (−3.4 to 3.2)	.94
**Age group: 12-14 mo**						
No.	277	419	302	NA	NA	NA
Underweight (WA *z* score <−2), No. (%)	70 (25.3)	105 (25.1)	89 (29.5)	−0.3 (−7.2 to 6.5)	5.5 (−2.8 to 13.7)	.25
Severely underweight (WA *z* score <−3), No.(%)	14 (5.1)	22 (5.3)	21 (7.0)	0.5 (−1.9 to 2.9)	2.6 (−0.5 to 5.7)	.21
Stunting (LA *z* score <−2), No. (%)	90 (32.5)	115 (27.4)	87 (28.8)	−5.9 (−13.9 to 2.0)	−3.8 (−13.1 to 5.5)	.32
Severe stunting (LA *z* score <−3), No. (%)	25 (9.0)	32 (7.6)	22 (7.3)	−0.9 (−5.3 to 3.4)	−0.2 (−5.5 to 5.1)	.89
Wasting (WL *z* score <−2), No. (%)	40 (14.4)	69 (16.5)	59 (19.5)	1.4 (−4.9 to 7.6)	6.6 (−1.6 to 14.8)	.24
Severe wasting (WL *z* score <−3), No. (%)	9 (3.2)	21 (5.0)	13 (4.3)	1.3 (−2.2 to 4.8)	1.0 (−3.1 to 5.0)	.76

Abbreviations: LA, length-for-age; NA, not applicable; pp, percentage points; WA, weight-for-age; WL, weight-for-length.

^a^ Data in the first 3 columns are descriptive and present observed numbers (proportions). Denominators (number per arm and age group) reflect the number of anthropometric measurements among children aged 6 to 8 months and 12 to 14 months pooled across visits; children who contributed more than 1 measurement within the same age band are counted separately for each measurement.

^b^ Adjusted estimates were obtained from mixed-effects models accounting for clustering at the village level and within-child correlation via random intercepts and adjusted for village size and child age group.

^c^ Overall comparison across intervention groups was conducted with the likelihood ratio test.

### Statistical Analysis

A mean of 7.5 children per village was expected to be measured, giving a total sample size of 1800 children (approximately 600 per trial group), providing 80% power to detect a 0.2-SD difference in anthropometric indices, consistent with effect sizes seen in nutritional supplementation trials. Sample size calculations for the parent trial are described elsewhere.^21^

We hypothesized that the mean weight, length, WA *z* score, LA *z* score, WL *z* score, and MUAC *z* score measured at 15, 18, 21, and 24 months after village enrollment would be higher among children aged 6 to 8 months and 12 to 14 months in villages receiving twice-yearly azithromycin compared with placebo villages, and likewise among those receiving quarterly azithromycin compared with placebo. We further hypothesized that the prevalence of underweight, stunting, and wasting would be lower in azithromycin groups compared with placebo.

Analyses were conducted at the participant level. Mean values for continuous outcomes were tabulated for each treatment arm using data pooled across measurement time points (15, 18, 21, and 24 months after village enrollment), with 95% CIs accounting for clustering by village. For categorical outcomes, numbers (proportions) were tabulated per treatment arm and age group (prespecified stratification) using data pooled across the same time points.

The effect of azithromycin MDA on growth outcomes was analyzed using mixed-effects regression models with random intercepts for clusters and for children (as a subset of children contributed repeated measurements across visits), adjusted for child age group (6-8 or 12-14 months) and the cluster-size stratification factor, based on national estimates, used in randomization (<100 infants or ≥100 infants). Continuous outcomes were modeled using linear mixed-effects models with an identity link, and categorical outcomes using mixed-effects logistic regression. A Kenward-Roger degrees of freedom correction was applied to all inferential tests, accounting for the small number of clusters in 1 arm.^26^ Overall comparisons across the 3 intervention groups were assessed using likelihood ratio tests, to control inflation of type I error across multiple treatment arms; the corresponding *P* values are reported. Pairwise contrasts (differences in mean values for continuous outcomes and differences in proportions for binary outcomes) are presented with corresponding 95% CIs. As a sensitivity analysis, all models were repeated without adjustment for age group or cluster size strata. Analyses were 2-sided, with an α level of .05 considered statistically significant.

Prespecified exploratory subgroup analyses evaluated the potential effect modification by child’s sex, age, and WA *z* score at enrollment; age group at anthropometric assessment; contextual factors including seasonality; receipt of seasonal malaria chemoprevention; round of MDA; distance to the nearest health facility; household asset index; water, sanitation, and hygiene index; and national outreach strategy (predefined interaction significance level of *P* < .10).

Missing anthropometric data arose primarily from unlinked measurements due to identifier mismatches. No imputation was performed, and analyses were conducted using complete cases. Analyses were performed in R, version 4 (R Project for Statistical Computing).

## Results

At baseline, a total of 1205 infants were enrolled: 341 from villages assigned to placebo, 494 from villages assigned to twice-yearly azithromycin, and 370 from villages assigned to quarterly azithromycin (Table 1). The mean age of the infants was 5.7 months (95% CI, 5.6-5.9 months); 633 (52.5%) were boys, 572 (47.5%) were girls, and 275 (22.8%) were underweight. Baseline characteristics were similar across groups.

**Figure 1.**
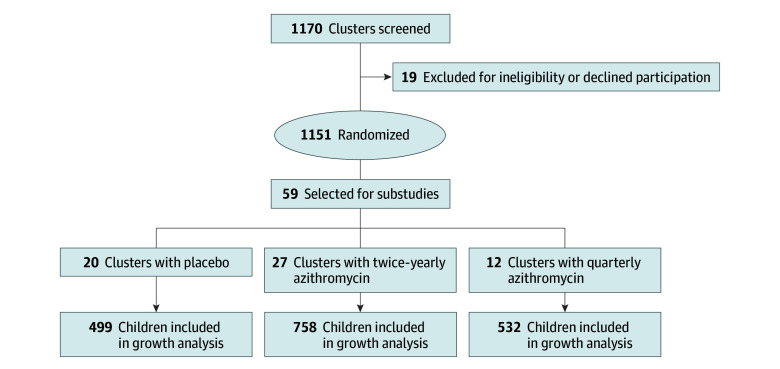
Study Flow Diagram There were 1151 villages (clusters) in southern Mali enrolled in the LAKANA (Large-Scale Assessment of the Key Health-Promoting Activities of Two New Mass Drug Administration Regimens With Azithromycin) placebo-controlled, 3-arm cluster randomized trial. Villages were randomized in a 3:4:2 ratio to receive placebo, twice-yearly azithromycin, or quarterly azithromycin. From these, 59 villages in the Kita region were selected to form the trial’s secondary outcome sample. Anthropometric data were collected at 4 quarterly visits from children aged 6 to 8 months and 12 to 14 months. Each age group represented a distinct cohort at each visit, although some children contributed data in both age groups at different time points. The number of clusters randomized and allocated to each arm (top) and the number of children included in the growth analysis (bottom) are displayed. Detailed counts of measurements at each visit, including valid and invalid or missing observations, are presented in eFigure 2 in Supplement 2.

Fifty-nine villages were included in this study: 20 received placebo, 27 received azithromycin 2 times a year, and 12 received azithromycin 4 times a year (Figure 1). All villages received their assigned intervention and remained in follow-up. Among children aged 6 to 8 months, 58.2% (96 of 165) to 90.8% (69 of 76) across study visits had received at least 1 prior dose of azithromycin by the time of a growth assessment (eTable 1 in Supplement 2). Among children aged 12 to 14 months, coverage ranged from 89.2% (66 of 74) to 97.6% (80 of 82).

Across all anthropometric assessments, invalid or unlinked measurements represented 25 of 633 assessments (4.0%) in the placebo arm, 49 of 940 (5.2%) in the twice-yearly azithromycin arm, and 25 of 658 (3.8%) in the quarterly azithromycin arm (eFigure 2 in Supplement 2). A total of 1789 children (499 in the placebo group, 758 in the twice-yearly azithromycin group, and 532 in the quarterly azithromycin group; 923 boys [51.6%] and 866 girls [48.4%]) contributed anthropometric data. Among these, 327 children (103 in the placebo group, 132 in the twice-yearly group, and 92 in the quarterly group) had repeated measurements across time points.

**Figure 2.**
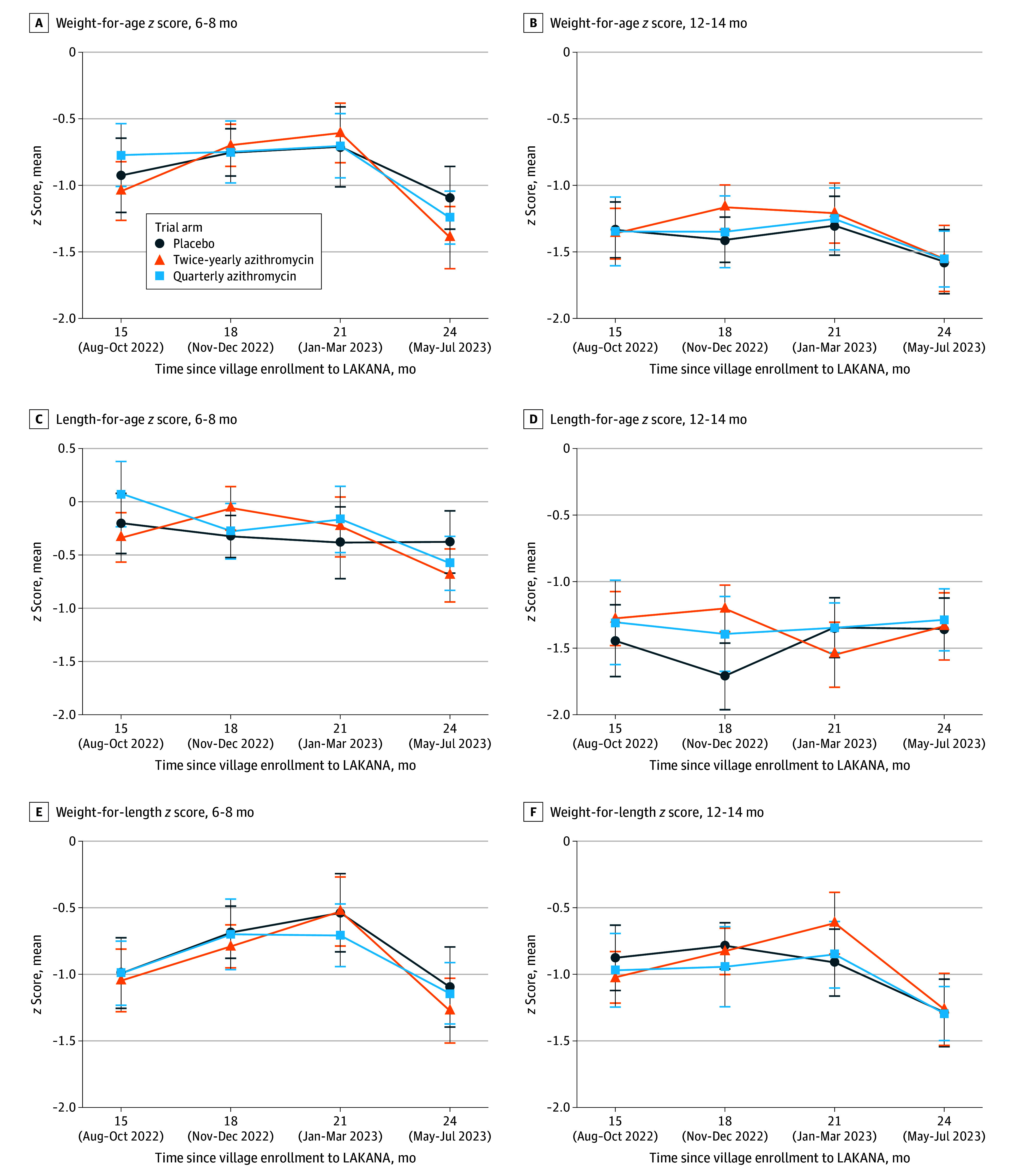
Line Graphs Showing Anthropometric Indices by Visit and Trial Arm Anthropometric indices over time by treatment group among children aged 6 to 8 months and 12 to 14 months who received azithromycin or placebo between 1 and 11 months of age. A total of 1789 children contributed valid anthropometric data across all visits. Data are presented as means at the time of assessment, with the y-axis showing the respective *z* score and the x-axis showing visit timing (time since village enrollment into the LAKANA [Large-Scale Assessment of the Key Health-Promoting Activities of Two New Mass Drug Administration Regimens With Azithromycin] trial and corresponding calendar months). Error bars indicate 95% CIs.

Based on MUAC screening, acute malnutrition was identified in 36 of 608 measurements in the placebo group (5.9%), 56 of 891 measurements in the twice-yearly azithromycin group (6.3%), and 33 of 633 measurements in the quarterly azithromycin group (5.2%). Among infants aged 6 to 8 months, the mean WA, LA, and WL *z* scores (Figure 2A, C, and E) were similar across groups over time. Among those aged 12 to 14 months, the mean values were also comparable across groups (Figure 2B, D, and F). The mean values for ponderal indices increased slightly from the first growth monitoring visit in late 2022 through the third in early 2023, then decreased at the fourth visit (from May to July 2023).

The mean weight and length and the mean WA, LA, WL, and MUAC *z* scores, pooled across the 4 monitoring visits, did not differ significantly between the placebo, twice-yearly azithromycin, and quarterly azithromycin groups: weight, 7.8 kg (95% CI, 7.7-7.9 kg), 7.8 kg (95% CI, 7.7-7.9 kg), and 7.8 kg (95% CI, 7.7-7.9 kg); length, 70.2 cm (95% CI, 69.9-70.8 cm), 70.6 cm (95% CI, 70.2-70.9 cm), and 70.6 cm (95% CI, 70.1-71.0 cm); WA *z* score, –1.12 (95% CI, −1.23 to −0.95), −1.10 (95% CI, −1.19 to −0.98), and −1.12 (95% CI, −1.27 to −1.01); LA *z* score, −0.86 (95% CI, −1.06 to −0.70), –0.79 (95% CI, −0.93 to −0.65), and –0.78 (95% CI, −0.98 to −0.63); WL *z* score, –0.87 (95% CI, −1.00 to −0.68), −0.89 (95% CI, −1.01 to −0.77), and −0.95 (95% CI, −1.12 to −0.81); and MUAC *z* score, −0.30 (95% CI, −0.43 to −0.10), −0.24 (95% CI, −0.37 to −0.12), and −0.26 (95% CI, −0.41 to −0.10), respectively (prespecified outcomes) (Table 2). The proportions of underweight, stunting, and wasting also did not differ significantly among the trial arms in either age group (Table 3). A sensitivity analysis without adjustment for cluster size and age group yielded similar results (eTables 2 and 3 in Supplement 2).

Among children with repeated measurements, the mean weight gain was 5.1 g/d (95% CI, 4.6-9.4 g/d) in the placebo group, 4.4 g/d (95% CI, 3.9-7.8 g/d) in the twice-yearly azithromycin group, and 4.3 g/d (95% CI, 3.1-7.6 g/d) in the quarterly azithromycin group (eTable 4 in Supplement 2). The mean length gain was 0.3 mm/d (95% CI, 0.3-0.4 mm/d) in the placebo group, 0.3 mm/d (95% CI, 0.3-0.4 mm/d) in the twice-yearly azithromycin group, and 0.3 mm/d (95% CI, 0.3-0.4 mm/d) in the quarterly azithromycin group; no statistically significant differences were observed across the 3 intervention groups (eTables 4 and 5 in Supplement 2). No clear evidence of effect modification of azithromycin MDA was observed across individual-level factors (sex, age, and baseline WA *z* score) or contextual level factors such as season; distance to the nearest health facility; household asset index; water, sanitation, and hygiene index; or exposure to seasonal malaria chemoprevention (eFigure 3a-d and eFigure 4a-d in Supplement 2).

## Discussion

In this analysis of secondary outcomes of a cluster randomized clinical trial in Mali, we evaluated the effects of azithromycin MDA targeted to infants aged 1 to 11 months on growth outcomes assessed at 6 to 8 months and 12 to 14 months of age. In a sample of 1789 children monitored at 15, 18, 21, and 24 months after community enrollment across 59 villages, neither the twice-yearly nor the quarterly azithromycin MDA regimens significantly affected weight and length or WA, LA, WL, and MUAC *z* scores compared with placebo. The proportion of underweight, stunting, and wasting also did not significantly differ between groups. Among a subset of children with repeated anthropometric measurements, no significant differences between groups were observed in the mean rate of weight or length gain. There was no evidence of effect modification by prespecified individual and contextual factors.

Our findings are consistent with prior studies of azithromycin MDA for trachoma control or child survival in sub-Saharan Africa, which have reported no overall growth benefits.^11,12,13,14,15,17,18^ The PRET (Partnership for Rapid Elimination of Trachoma)^11^ and MORDOR (Macrolides Oraux pour Réduire les Décès avec un Oeil sur la Résistance)^12^ trials in Niger and the TANA (Trachoma Amelioration in Northern Amhara)^13^ trial in Ethiopia, all of which reported child survival benefits, found no overall growth benefits of biannual mass azithromycin distribution among children younger than 5 years. However, in the MORDOR trial, among those in the lowest quartile of baseline height, children randomized to the azithromycin group were approximately 0.4 cm taller at 4 years.^12^ In Burkina Faso, the NAITRE (Nouveux-nés et Azithromycine: une Innovation dans le Traitement des Enfants) and CHATON (Child Health With Azithromycin Trial) trials, which delivered single-dose azithromycin to infants aged 1 to 12 weeks, showed no improvement in growth by 6 months,^14,15^ and a long-term follow-up of NAITRE participants found no differences at 4 years.^17^ A subgroup analysis of the NAITRE trial found that neonates who had low birth weight (<2500 g) and were underweight (WA *z* score <−2) at enrollment had lower odds of being underweight at 6 months if they received azithromycin compared with placebo.^16^ However, a pooled secondary analysis^18^ of the NAITRE and CHATON trials found no evidence that azithromycin administered in early infancy prevents underweight, wasting, or stunting among infants classified by WHO as at risk of poor growth and development (WA *z* score <−2, WL *z* score <−2, or MUAC <110 mm). In our study, we similarly found no differential treatment effect among infants with a low WA *z* score at enrollment. Unlike prior trials, we assessed both 2- and 4-dose regimens administered quarterly during infancy, providing additional evidence that neither cumulative exposure nor increased dosing frequency influences growth.

Mortality reductions in West African settings have been observed when azithromycin is administered to children aged 1 to 59 months, but not when restricted to infants aged 1 to 11 months,^5^ as in the LAKANA trial.^24^ One possible explanation is that older children play a larger role in sustaining infectious disease transmission.^27^ If growth benefits of azithromycin are primarily mediated through reductions in infection burden in the community, then the absence of significant improvements in infant anthropometry is consistent with the lack of mortality benefit observed when treatment is restricted to infants. Beyond infection control, azithromycin may influence growth by altering the gut microbiota, reducing inflammation and enhancing nutrient absorption, although research on these pathways is ongoing. Reducing inflammation may also restore insulin-like growth factor 1 (IGF-1), a key regulator of linear growth.^28^ IGF-1 plays an important role in fetal growth, but growth during early infancy is primarily nutrition dependent and mediated largely through insulin, thyroid hormones, and growth hormone (GH)–independent IGF-1 pathways, with GH-induced IGF-1 signaling becoming increasingly important from midinfancy onward.^28,29,30,31^ This developmental shift suggests that the younger infants in our trial, whose growth remains largely nutrition driven, may be less responsive to interventions possibly acting through inflammation and GH-induced IGF-1–related pathways, which may have contributed to the lack of detectable growth effects. Moreover, the ELICIT (Early Life Interventions for Childhood Growth and Development In Tanzania) individually randomized clinical trial found no growth benefit from scheduled azithromycin and nitazoxanide administration among children aged 18 months or younger.^32^ Because the ELICIT trial was conducted in a setting with a high prevalence of stunting and enteric pathogen carriage, the authors suggest that chronic dietary deficits and other disease burdens may play a dominant role in restricting growth in such environments. In the ELICIT trial, reductions observed 2 weeks after dosing were no longer evident at 3 months, suggesting rapid recolonization in settings of ongoing exposure, and may also explain the absence of detectable or durable improvements in growth.

### Limitations

This study has some limitations. The selection of the secondary outcomes’ sample based on proximity to health facilities may limit generalizability. However, given the rural and relatively homogeneous trial setting, major differences are unlikely. Although no effect modification by rainy vs nonrainy season was detected, the timing of visits across lean and postharvest periods, together with year-to-year variability, may have reduced our ability to distinguish seasonal influences from intervention effects.^33,34^ Although prespecified, the study was not powered for subgroup analyses; these analyses were intended to provide preliminary signals that warrant confirmation in larger studies.

Cluster size varied across trial arms, with a few large villages in the azithromycin groups and none in the placebo group. Although mixed-effects models accounted for clustering, this imbalance may have contributed to additional variability in the effect estimates. Overall comparisons across the 3 arms used likelihood ratio tests to control type I error inflation; however, this approach does not adjust for multiplicity across the several secondary outcomes examined. The analyses should therefore be interpreted as hypothesis generating.

Children were assessed cross-sectionally rather than longitudinally. Nevertheless, randomization preserved comparability between groups, and the repeated-measures subset enabled the preliminary assessment of individual-level changes over time. As the trial involved infants younger than 1 year, findings may not be generalizable to older children. Future studies including older age groups or with extended follow-up may provide additional insights into the potential growth effects of azithromycin. Finally, the study was conducted in regions less affected by conflict than northern Mali, where access to health services is more limited and food insecurity and infectious disease burden are higher,^35^ which may limit generalizability to more vulnerable populations.

## Conclusions

In this analysis of secondary outcomes of a cluster randomized clinical trial of azithromycin mass administration to infants 1 to 11 months of age in Mali, growth outcomes did not differ between the azithromycin and placebo groups. These findings suggest that mass administration of azithromycin to infants is unlikely to promote growth.

## Notes

Supplement 1.
Trial Protocol and Statistical Analysis Plan

Supplement 2. **eTable 1. **Distribution of Azithromycin Doses Received Prior to Each Growth Assessment **eTable 2. **Anthropometric Outcomes by Treatment Group Among Children Aged 6–8 and 12–14 Months, Non-Adjusted Analysis **eTable 3.** Prevalence of Underweight, Stunting, and Wasting by Treatment Group and Age Group, Non-Adjusted Analysis **eTable 4. **Weight and Length Gains by Treatment Group Among Children Aged 6–8 and 12–14 Months With Repeated Measurements **eTable 5. **Weight and Length Gains by Treatment Group Among Children Aged 6–8 and 12–14 Months With Repeated Measurements, Non-Adjusted Analysis **eFigure 1. **Timeline for the Trial Mass Drug Administration Rounds (MDA) and the Growth Substudy Visits **eFigure 2. **Detailed Study Flow and Accounting of Child-Level Anthropometric Measurements Across Quarterly Visits **eFigure 3a. **Mean Differences in Weight-for-Age z Scores (WAZ) Between Control and Quarterly Azithromycin Groups by Child and Household Characteristics **eFigure 3b. **Mean Differences in Length-for-Age z Scores (LAZ) Between Control and Quarterly Azithromycin Groups by Child and Household Characteristics **eFigure 3c. **Mean Differences in Weight-for-Length z Scores (WLZ) Between Control and Quarterly Azithromycin Groups by Child and Household Characteristics **eFigure 3d. **Mean Differences in Mid-Upper-Arm Circumference z Scores (MUACZ) Between Control and Quarterly Azithromycin Groups by Child and Household Characteristics **eFigure 4a. **Mean Differences in Weight-for-Age z Scores (WAZ) Between Control and Quarterly Azithromycin Groups by Ecological Characteristics **eFigure 4b. **Mean Differences in Length-for-Age z Scores (LAZ) Between Control and Quarterly Azithromycin Groups by Ecological Characteristics **eFigure 4c. **Mean Differences in Weight-for-Length z Scores (WLZ) Between Control and Quarterly Azithromycin Groups by Ecological Characteristics **eFigure 4d. **Mean Differences in Mid-Upper-Arm Circumference z Scores (MUACZ) Between Control and Quarterly Azithromycin Groups by Ecological Characteristics

Supplement 3.
Data Sharing Statement
