# Supplementary material for: Infant Growth After Mass Administration of Azithromycin: Secondary Outcomes of a Cluster Randomized Clinical Trial
Source: JAMA Netw Open. 2026 Jun 10;9(6):e2617425. doi: 10.1001/jamanetworkopen.2026.17425 (PMC13254738; doi:10.1001/jamanetworkopen.2026.17425)
Supplement: Supplement 2. — eTable 1. Distribution of Azithromycin Doses Received Prior to Each Growth Assessment eTable 2. Anthropometric Outcomes by Treatment Group Among Children Aged 6–8 and 12–14 Months, Non-Adjusted Analysis eTable 3. Prevalence of Underweight, Stunting, and Wasting by Treatment Group and Age Group, Non-Adjusted Analysis eTable 4. Weight and Length Gains by Treatment Group Among Children Aged 6–8 and 12–14 Months With Repeated Measurements eTable 5. Weight and Length Gains by Treatment Group Among Children Aged 6–8 and 12–14 Months With Repeated Measurements, Non-Adjusted Analysis eFigure 1. Timeline for the Trial Mass Drug Administration Rounds (MDA) and the Growth Substudy Visits eFigure 2. Detailed Study Flow and Accounting of Child-Level Anthropometric Measurements Across Quarterly Visits eFigure 3a. Mean Differences in Weight-for-Age z Scores (WAZ) Between Control and Quarterly Azithromycin Groups by Child and Household Characteristics eFigure 3b. Mean Differences in Length-for-Age z Scores (LAZ) Between Control and Quarterly Azithromycin Groups by Child and Household Characteristics eFigure 3c. Mean Differences in Weight-for-Length z Scores (WLZ) Between Control and Quarterly Azithromycin Groups by Child and Household Characteristics eFigure 3d. Mean Differences in Mid-Upper-Arm Circumference z Scores (MUACZ) Between Control and Quarterly Azithromycin Groups by Child and Household Characteristics eFigure 4a. Mean Differences in Weight-for-Age z Scores (WAZ) Between Control and Quarterly Azithromycin Groups by Ecological Characteristics eFigure 4b. Mean Differences in Length-for-Age z Scores (LAZ) Between Control and Quarterly Azithromycin Groups by Ecological Characteristics eFigure 4c. Mean Differences in Weight-for-Length z Scores (WLZ) Between Control and Quarterly Azithromycin Groups by Ecological Characteristics eFigure 4d. Mean Differences in Mid-Upper-Arm Circumference z Scores (MUACZ) Between Control and Quarterly Azithromycin Groups by Ecological Character [file jamanetwopen-e2617425-s002.pdf]

Supplementary Online Content

Adubra L, Luoma J, Fan YM, et al. Infant growth after mass administration of azithromycin: secondary outcomes of a cluster randomized clinical trial. *JAMA Netw Open.* 2026;9(6):e2617425. doi:10.1001/jamanetworkopen.2026.17425

eTable 1. Distribution of Azithromycin Doses Received Prior to Each Growth Assessment
eTable 2. Anthropometric Outcomes by Treatment Group Among Children Aged 6–8 and 12–14 Months, Non-Adjusted Analysis
eTable 3. Prevalence of Underweight, Stunting, and Wasting by Treatment Group and Age Group, Non-Adjusted Analysis
eTable 4. Weight and Length Gains by Treatment Group Among Children Aged 6–8 and 12–14 Months With Repeated Measurements
eTable 5. Weight and Length Gains by Treatment Group Among Children Aged 6–8 and 12–14 Months With Repeated Measurements, Non-Adjusted Analysis
eFigure 1. Timeline for the Trial Mass Drug Administration Rounds (MDA) and the Growth Substudy Visits
eFigure 2. Detailed Study Flow and Accounting of Child-Level Anthropometric Measurements Across Quarterly Visits
eFigure 3a. Mean Differences in Weight-for-Age z Scores (WAZ) Between Control and Quarterly Azithromycin Groups by Child and Household Characteristics
eFigure 3b. Mean Differences in Length-for-Age z Scores (LAZ) Between Control and Quarterly Azithromycin Groups by Child and Household Characteristics
eFigure 3c. Mean Differences in Weight-for-Length z Scores (WLZ) Between Control and Quarterly Azithromycin Groups by Child and Household Characteristics
eFigure 3d. Mean Differences in Mid-Upper-Arm Circumference z Scores (MUACZ) Between Control and Quarterly Azithromycin Groups by Child and Household Characteristics
eFigure 4a. Mean Differences in Weight-for-Age z Scores (WAZ) Between Control and Quarterly Azithromycin Groups by Ecological Characteristics
eFigure 4b. Mean Differences in Length-for-Age z Scores (LAZ) Between Control and Quarterly Azithromycin Groups by Ecological Characteristics
eFigure 4c. Mean Differences in Weight-for-Length z Scores (WLZ) Between Control and Quarterly Azithromycin Groups by Ecological Characteristics
eFigure 4d. Mean Differences in Mid-Upper-Arm Circumference z Scores (MUACZ) Between Control and Quarterly Azithromycin Groups by Ecological Characteristics

This supplementary material has been provided by the authors to give readers additional information about their work.

© 2026 Adubra L et al. *JAMA Network Open.*

eTable 1. Distribution of Azithromycin Doses Received Prior to Each Growth Assessment[a]

Growth visit (Corresponding trial round)	Child age group at growth visit	Arm	N	Mean Number of AZI Doses Received (up to visit)	0 dose n (%)	1 dose n (%)	2 doses n (%)	3 doses n (%)	4 doses n (%)	Coverage ≥1 dose (%)
1 (MDA 6)	12-14 months	Twice-yearly AZI	132	1.6	7 (5.3%)	39 (29.5%)	86 (65.2%)	—[b]	—[b]	94.7
1 (MDA 6)	6-8 months	Twice-yearly AZI	107	1.12	19 (17.8%)	56 (52.3%)	32 (29.9%)	—	—	82.2
2 (MDA 7)	12-14 months	Twice-yearly AZI	141	1.58	8 (5.7%)	43 (30.5%)	90 (63.8%)	—	—	94.3
2 (MDA 7)	6-8 months	Twice-yearly AZI	165	0.59	69 (41.8%)	94 (57.0%)	2 (1.2%)	—	—	58.2
3 (MDA 8)	12-14 months	Twice-yearly AZI	93	1.47	10 (10.8%)	29 (31.2%)	54 (58.1%)	—	—	89.2
3 (MDA 8)	6-8 months[c]	Twice-yearly AZI	87	—	—	—	—	—	—	—
4 (Close out visit)	12-14 months	Twice-yearly AZI	74	1.31	8 (10.8%)	35 (47.3%)	31 (41.9%)	—	—	89.2
4 (Close out visit)	6-8 months	Twice-yearly AZI	93	0.71	27 (29.0%)	66 (71.0%)	0 (0.0%)	—	—	71
1 (MDA 6)	12-14 months	Quarterly AZI	82	2.2	2 (2.4%)	15 (18.3%)	30 (36.6%)	35 (42.7%)	0 (0.0%)	97.6
1 (MDA 6)	6-8 months[d]	Quarterly AZI	62	1.05	9 (14.5%)	41 (66.1%)	12 (19.4%)	0 (0.0%)	0 (0.0%)	85.5
2 (MDA 7)	12-14 months	Quarterly AZI	76	2.29	3 (3.9%)	10 (13.2%)	30 (39.5%)	28 (36.8%)	5 (6.6%)	96.1
2 (MDA 7)	6-8 months[d]	Quarterly AZI	98	1.29	18 (18.4%)	35 (35.7%)	44 (44.9%)	1 (1.0%)	0 (0.0%)	81.6
3 (MDA 8)	12-14 months	Quarterly AZI	67	2.84	2 (3.0%)	3 (4.5%)	15 (22.4%)	31 (46.3%)	16 (23.9%)	97
3 (MDA 8)	6-8 months[d]	Quarterly AZI	76	1.46	7 (9.2%)	31 (40.8%)	34 (44.7%)	4 (5.3%)	0 (0.0%)	90.8
4 (Close out visit)	12-14 months	Quarterly AZI	86	2.59	4 (4.7%)	8 (9.3%)	25 (29.1%)	31 (36.0%)	18 (20.9%)	95.3
4 (Close out visit)	6-8 months	Quarterly AZI	86	1.06	20 (23.3%)	41 (47.7%)	25 (29.1%)	0 (0.0%)	0 (0.0%)	76.7

[a]Infants aged 1–11 months in LAKANA Trial were eligible to receive oral azithromycin (AZI) during mass-drug-administration (MDA) rounds held every 3 months. In the quarterly arm, children could receive azithromycin at every visit, whereas in the biannual arm they received azithromycin only at the Jan–Jun visits each year.

[b]— Not applicable by design

[c]Because the prior MDA rounds 6–7 were placebo in the biannual arm, only MDA 5 could contribute prior azithromycin before the growth visit 3; Infants aged 6-8 months were not age-eligible for treatment at MDA 5.

[d]In the quarterly arm, infants at the upper end of the 6–8-month band could have been age-eligible for azithromycin at more than 2 prior rounds, particularly in villages where these rounds occurred earlier than planned. Consequently, a small proportion accrued up to three prior doses.

© 2026 Adubra L et al. *JAMA Network Open.*

eTable 2. Anthropometric Outcomes by Treatment Group Among Children Aged 6–8 and 12–14 Months, Non-Adjusted Analysis[a]

	Control (n = 499)	Twice-yearly azithromycin (n = 758)	Quarterly azithromycin (n = 532)	Difference in means (95% CI)[b,c] Twice-yearly azithromycin vs Control	Difference in means (95% CI) Quarterly azithromycin vs Control
Weight, mean[b] (95%CI), kg	7.77 (7.68 to 7.94)	7.83 (7.74 to 7.93)	7.80 (7.68 to 7.91)	0.06 (-0.10 to 0.21)	-0.03 (-0.16 to 0.21)
Length, mean (95%CI), cm	70.2 (69.9 to 70.8)	70.6 (70.2 to 70.9)	70.6 (70.1 to 71.0)	0.35 (-0.27 to 0.98)	0.42 (-0.33 to 1.17)
WAZ, mean (95%CI)	-1.12 (-1.23 to -0.95)	-1.10 (-1.19 to -0.98)	-1.12 (-1.27 to -1.01)	0.01 (-0.15 to 0.18)	-0.03 (-0.23 to 0.16)
LAZ, mean (95%CI)	-0.86 (-1.06 to -0.70)	-0.79 (-0.93 to -0.65)	-0.78 (-0.98 to -0.63)	0.07 (-0.13 to 0.28)	0.06 (-0.18 to 0.30)
WLZ, mean (95%CI)	-0.87 (-1.00 to -0.68)	-0.89 (-1.01 to -0.77)	-0.95 (-1.12 to -0.81)	-0.03 (-0.22 to 0.14)	-0.11 (-0.32 to 0.11)
MUACZ, mean (95%CI)	-0.3 (-0.43 to -0.10)	-0.2 (-0.37 to -0.12)	-0.3 (-0.41 to -0.10)	0.02 (-0.15 to 0.19)	0.01 (-0.20 to 0.22)

LAZ: length-for-age Z-score, MUAC: mid-upper-arm circumference, WAZ: weight-for-age Z-score, WLZ: weight-for-length Z-score.

[a]Data in the first three columns present individual-level observed means based on anthropometric measurements pooled across the 15-, 18-, 21-, and 24-month visits; 95% confidence intervals account for clustering by village. The n per arm reflects the number of unique children who contributed at least one measurement; some children contributed repeated measurements at different visits.

[b]Mixed-effects models used for inference included random intercepts for village and child to account for clustering and within-child correlation.

[c]Overall comparison across intervention groups (likelihood ratio test): weight (P =.68), length (P =.31), WAZ (P = .80), LAZ (P = .67), WLZ (P = .48), MUACZ (P = .98).

© 2026 Adubra L et al. *JAMA Network Open.*

eTable 3. Prevalence of Underweight, Stunting, and Wasting by Treatment Group and Age Group, Non-Adjusted Analysis[a]

	Control	Twice-yearly azithromycin	Quarterly azithromycin	Difference in proportions, pp (95% CI)[b,c] Twice-yearly azithromycin vs Control	Difference in proportions, pp (95% CI)[b,c] Quarterly azithromycin vs Control
Age Group: 6-8 months	n = 300	n = 422	n = 304		
Underweight (WAZ <-2), No. (%)	50 (16.7)	76 (18.0)	44 (14.5)	1.21 (-4.13 to 6.54)	-1.92 (-7.46 to 3.61)
Severely underweight(WAZ <-3),No.(%)	10 (3.3)	19 (4.5)	10 (3.3)	1.01 (-1.87 to 3.89)	-0.01 (-3.04 to 3.02)
Stunted (LAZ <-2), No. (%)	26 (8.7)	39 (9.2)	30 (9.9)	0.52 (-3.48 to 4.51)	1.19 (-3.21 to 5.60)
Severely stunted (LAZ <-3), No. (%)	4 (1.3)	6 (1.4)	8 (2.6)	0.08 (-1.53 to 1.69)	1.24 (-0.85 to 3.33)
Wasted (WLZ <-2), No. (%)	48 (16.0)	73 (17.3)	54 (17.8)	1.02 (-4.65 to 6.68)	2.30 (-4.33 to 8.92)
Severely wasted (WLZ <-3), No. (%)	7 (2.3)	13 (3.1)	10 (3.3)	0.67 (-1.55 to 2.90)	0.85 (-1.62 to 3.32)
Age Group: 12-14 months	n = 277	n = 419	n = 302		
Underweight (WAZ <-2), No. (%)	70 (25.3)	105 (25.1)	89 (29.5)	-0.97 (-8.02 to 6.08)	4.64 (-3.65 to 12.93)
Severely underweight(WAZ <-3),No.(%)	14 (5.1)	22 (5.3)	21 (7.0)	0.09 (-3.31 to 3.49)	2.09 (-2.10 to 6.28)
Stunted (LAZ <-2), No. (%)	90 (32.5)	115 (27.4)	87 (28.8)	-5.79 (-13.4 to 1.81)	-3.61 (-12.3 to 5.03)
Severely stunted (LAZ <-3), No. (%)	25 (9.0)	32 (7.6)	22 (7.3)	-1.68 (-6.88 to 3.52)	-1.30 (-7.35 to 4.76)
Wasted (WLZ <-2), No. (%)	40 (14.4)	69 (16.5)	59 (19.5)	1.10 (-5.37 to 7.57)	6.31 (-2.14 to 14.76)
Severely wasted (WLZ <-3), No. (%)	9 (3.2)	21 (5.0)	13 (4.3)	1.43 (-1.73 to 4.58)	1.20 (-2.45 to 4.84)

LAZ: length-for-age Z-score, MUAC: mid-upper-arm circumference, WAZ: weight-for-age Z-score, WLZ: weight-for-length Z-score, pp: percentage points.

[a]Data in the first three columns are descriptive and present observed numbers (proportions). Denominators (n per arm and age group) reflect the number of anthropometric measurements among children aged 6–8 months and 12–14 months pooled across visits; children who contributed more than one measurement within the same age band are counted separately for each measurement.

[b]Mixed-effects models used for inference accounted for clustering at village level and within child correlation via random intercepts.

[c]Overall comparison across intervention groups (likelihood ratio test): Age Group: 6-8 months: Underweight (P = .51), Severely Underweight (P = .72), Stunted (P = .86), Severely stunted (P = .39), Wasted (P = .78), Severely wasted (P = .76); Age Group: 12-14 months: Underweight (P = .30), Severely Underweight (P = .49), Stunted (P = .31), Severely stunted (P = .81), Wasted (P = .25), Severely wasted (P = .65).

© 2026 Adubra L et al. *JAMA Network Open.*

eTable 4. Weight and Length Gains by Treatment Group Among Children Aged 6–8 and 12–14 Months With Repeated Measurements[a]

	Placebo (n = 103)	Twice-yearly azithromycin (n = 132)	Quarterly azithromycin (n = 92)	Difference in means (95% CI)[b,c] Twice-yearly azithromycin vs Control	Difference in means (95% CI) Quarterly azithromycin vs Control
Weight gain, mean (95%CI), g/day	5.11 (4.56 to 9.44)	4.40 (3.86 to 7.76)	4.33 (3.08 to 7.60)	-1.19 (-3.97 to 1.59)	-1.66 (-5.00 to 1.68)
Length gain, mean (95%CI), mm/day	0.30 (0.27 to 0.42)	0.29 (0.27 to 0.39)	0.29 (0.25 to 0.39)	-0.02 (-0.10 to 0.07)	-0.02 (-0.13 to 0.08)

[a]Means are presented with 95% confidence intervals that account for clustering by village. The n per arm reflects the number of unique children who contributed repeated measurements across visits.
[b]Adjusted estimates were obtained from mixed-effects models accounting for clustering at the village level and within-child correlation via random intercepts and adjusted for village size and child age group.
[c]Overall comparison across intervention groups (likelihood ratio test): weight gain (P =.41), length gain (P =.83).

© 2026 Adubra L et al. *JAMA Network Open.*

eTable 5. Weight and Length Gains by Treatment Group Among Children Aged 6–8 and 12–14 Months With Repeated Measurements, Non-Adjusted Analysis[a]

	Placebo (n = 103)	Twice-yearly azithromycin (n = 132)	Quarterly azithromycin (n = 92)	Difference in means (95% CI)[b,c] Twice-yearly azithromycin vs Control	Difference in means (95% CI) Quarterly azithromycin vs Control
Weight gain, mean (95%CI), g/day	5.11 (4.56 to 9.44)	4.40 (3.86 to 7.76)	4.33 (3.08 to 7.60)	-0.74 (-3.45 to 1.98)	-0.92 (-4.09 to 2.25)
Length gain, mean (95%CI), mm/day	0.30 (0.27 to 0.42)	0.29 (0.27 to 0.39)	0.29 (0.25 to 0.39)	-0.01 (-0.09 to 0.08)	-0.01 (-0.11 to 0.09)

[a]Means are presented with 95% confidence intervals that account for clustering by village. The n per arm reflects the number of unique children who contributed repeated measurements across visits.

[b]Mixed-effects models used for inference included random intercepts for village and child to account for clustering and within-child correlation.

[c]Overall comparison across intervention groups (likelihood ratio test): weight gain (P =.72), length gain (P =.96).

© 2026 Adubra L et al. *JAMA Network Open.*

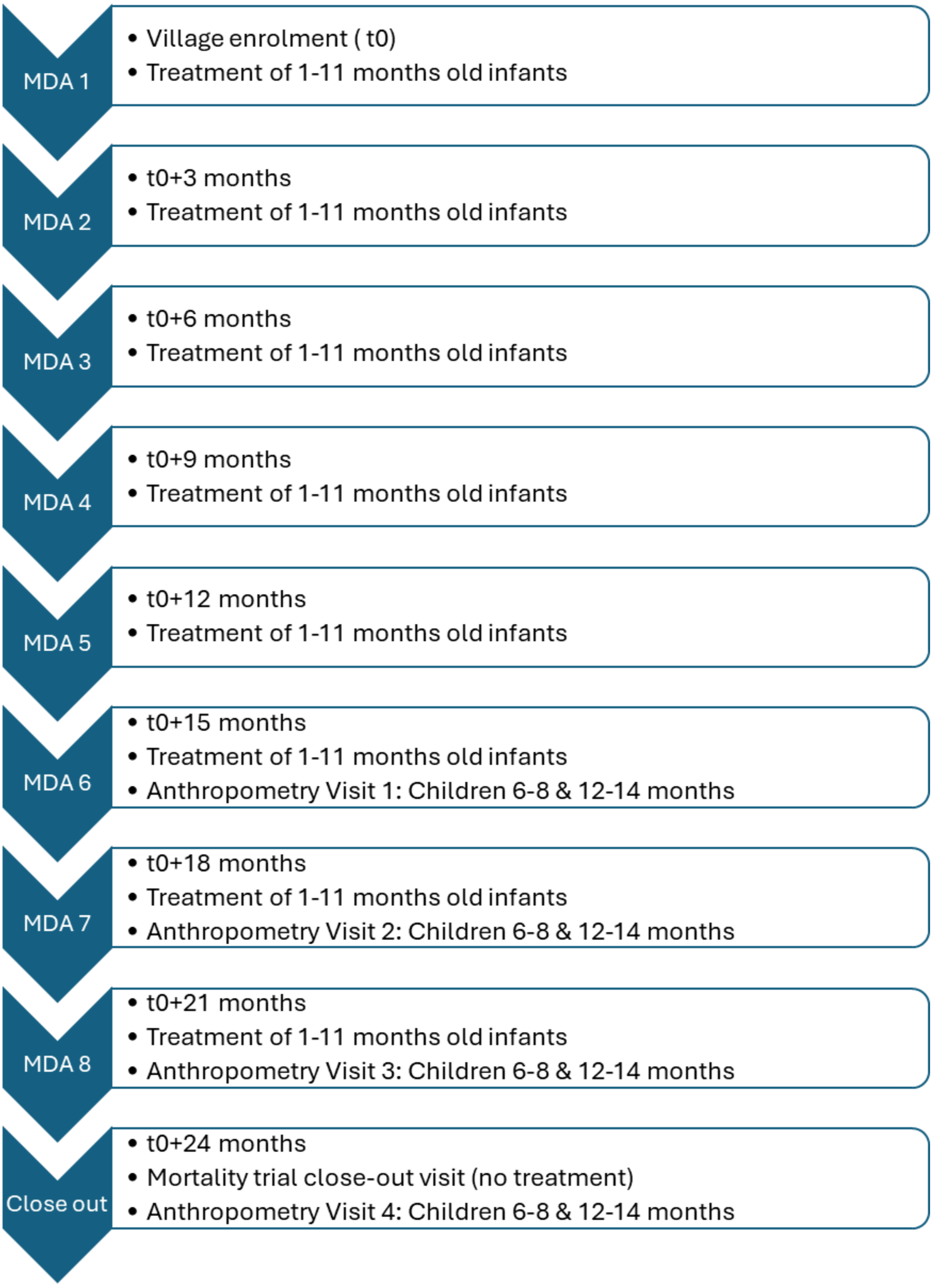

eFigure 1. Timeline for the Trial Mass Drug Administration Rounds (MDA) and the Growth Substudy Visits

© 2026 Adubra L et al. *JAMA Network Open.*

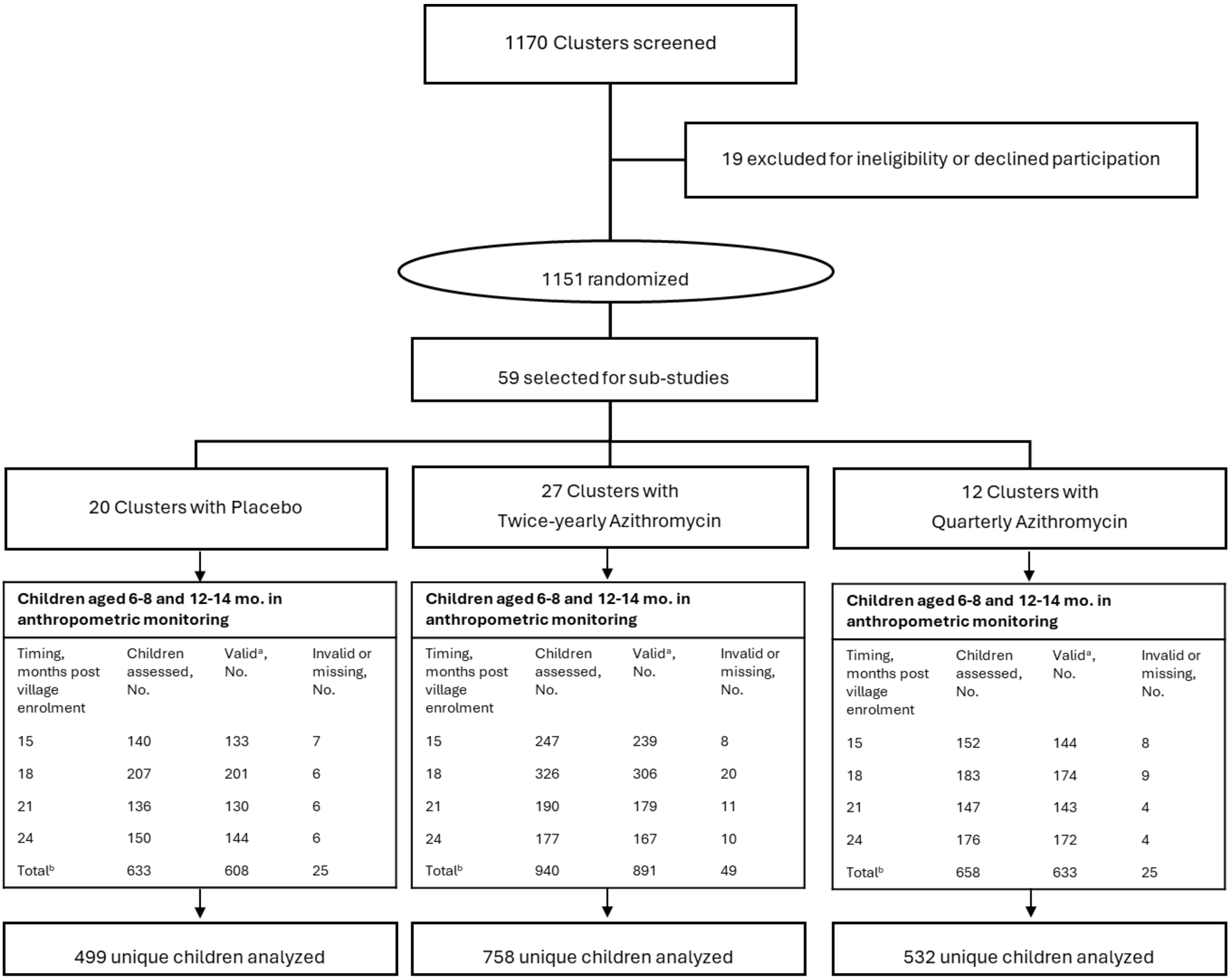

20 Clusters with Placebo — Children aged 6-8 and 12-14 mo. in anthropometric monitoring

Timing, months post village enrolment	Children assessed, No.	Valid[a], No.	Invalid or missing, No.
15	140	133	7
18	207	201	6
21	136	130	6
24	150	144	6
Total[b]	633	608	25

27 Clusters with Twice-yearly Azithromycin — Children aged 6-8 and 12-14 mo. in anthropometric monitoring

Timing, months post village enrolment	Children assessed, No.	Valid[a], No.	Invalid or missing, No.
15	247	239	8
18	326	306	20
21	190	179	11
24	177	167	10
Total[b]	940	891	49

12 Clusters with Quarterly Azithromycin — Children aged 6-8 and 12-14 mo. in anthropometric monitoring

Timing, months post village enrolment	Children assessed, No.	Valid[a], No.	Invalid or missing, No.
15	152	144	8
18	183	174	9
21	147	143	4
24	176	172	4
Total[b]	658	633	25

eFigure 2. Detailed Study Flow and Accounting of Child-Level Anthropometric Measurements Across Quarterly Visits

There were 1151 villages (clusters) in southern Mali enrolled in the LAKANA placebo-controlled, three-arm cluster-randomized trial. Villages were randomized in a 3:4:2 ratio to receive placebo, twice-yearly azithromycin, or quarterly azithromycin. From these, 59 villages in the Kita region were selected to form the trial's secondary outcome sample. Infants aged 1–11 months were eligible for study treatment, and anthropometric data were collected at four quarterly visits from children aged 6–8 and 12–14 months. Each age group represented a distinct cohort at each visit, although some children contributed data in both age groups at different time points.

The top section of the figure shows the number of clusters (villages), and the bottom shows the number of children in anthropometric monitoring. A total of 1,789 children contributed valid anthropometric data across all visits; 327 of these children had repeated measurements across multiple visits. Counts under 'children assessed' represent the total number of anthropometric measurements across the 15 , 18 , 21 , and 24 month visits. Because some children were measured at multiple visits, the total exceeds the number of unique children, which is shown in the 'children analyzed' row.

[a] Anthropometric indices (z-scores) within the World Health Organization (WHO) acceptable range.

[b] These totals reflect the number of measurements, not unique participants. Individuals contributing to multiple visits are counted at each visit.

© 2026 Adubra L et al. *JAMA Network Open.*

Note: Effect-modification analyses were conducted for both the twice-yearly and quarterly azithromycin groups. Results for the twice-yearly group were similar to those shown here and did not demonstrate evidence of effect modification; therefore, only the quarterly arm is presented.

eFigure 3a. Mean Differences in Weight-for-Age z Scores (WAZ) Between Control and Quarterly Azithromycin Groups by Child and Household Characteristics[a]

[a]Data were collected at mass drug administration (MDA) rounds 6–8 and the close-out visit (15, 18, 21, and 24 months after village enrollment). Infants received azithromycin or placebo between ages 1 and 11 months. Displayed values are observed (raw) group means ± SD. Estimates (mean difference, 95% CI) are shown for Azithromycin quarterly vs Placebo. *P* values are from global likelihood ratio tests of subgroup × intervention interaction including all study arms (Control, Twice-yearly, Quarterly). Confidence intervals extending beyond the axis range are truncated in the figure and shown with dotted lines.

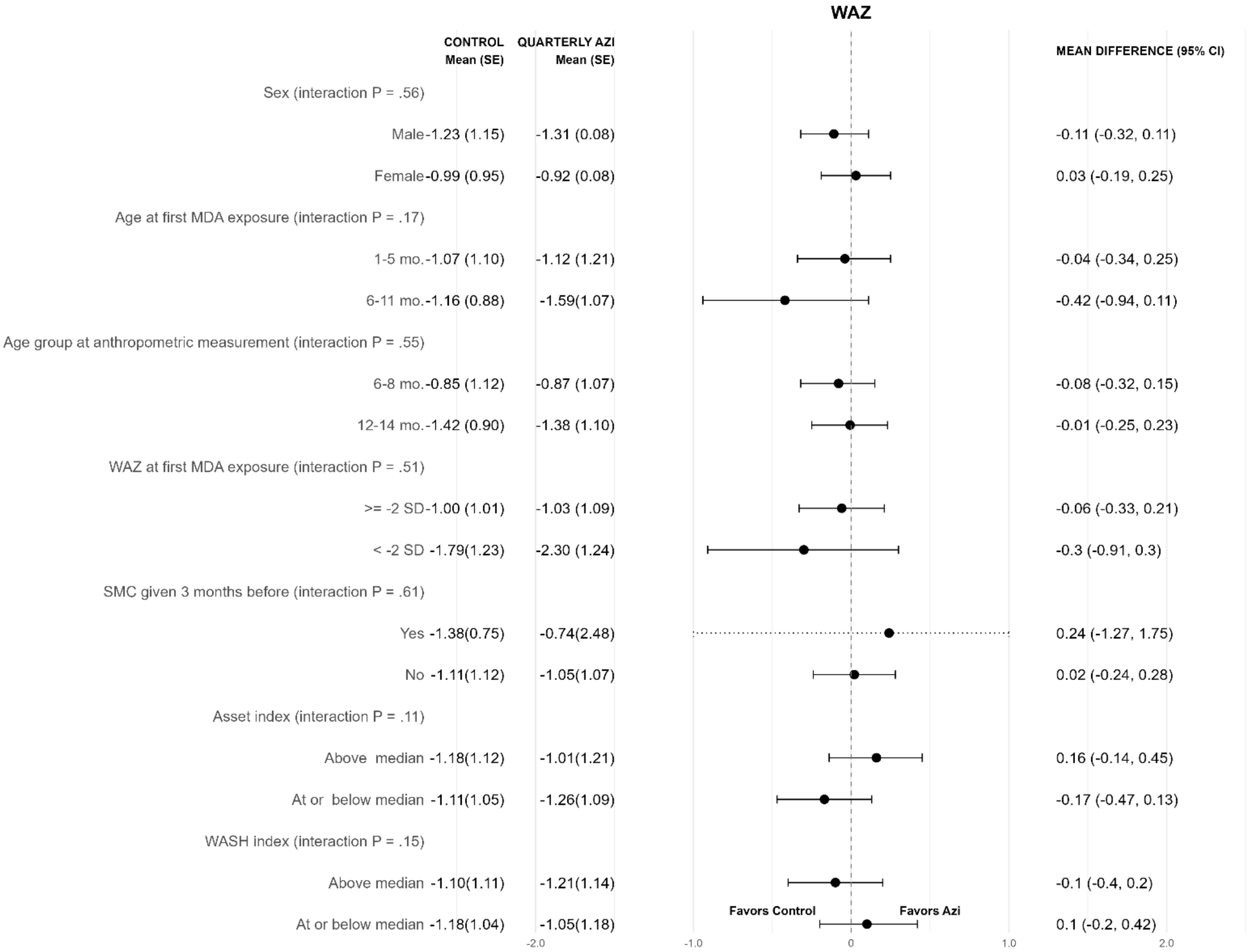

© 2026 Adubra L et al. *JAMA Network Open.*

eFigure 3b. Mean Differences in Length-for-Age z Scores (LAZ) Between Control and Quarterly Azithromycin Groups by Child and Household Characteristics[a]

[a]Data were collected at mass drug administration (MDA) rounds 6–8 and the close-out visit (15, 18, 21, and 24 months after village enrollment). Infants received azithromycin or placebo between ages 1 and 11 months. Displayed values are observed (raw) group means ± SD. Estimates (mean difference, 95% CI) are shown for Azithromycin quarterly vs Placebo. P values are from global likelihood ratio tests of subgroup × intervention interaction including all study arms (Control, Twice-yearly, Quarterly). Confidence intervals extending beyond the axis range are truncated in the figure and shown with dotted lines.

	CONTROL Mean (SE)	QUARTERLY AZI Mean (SE)	MEAN DIFFERENCE (95% CI)
Sex (interaction P = .70)			
Male	-1.02(1.28)	-0.93(1.37)	0.09 (-0.17, 0.37)
Female	-0.69(1.31)	-0.63(1.32)	0.06 (-0.21, 0.34)
Age at first MDA exposure (interaction P = .34)			
1-5 mo.	-0.84(1.28)	-0.66(1.47)	0.2 (-0.18, 0.58)
6-11 mo.	-1.42(1.28)	-1.57(1.18)	-0.14 (-0.75, 0.48)
Age group at anthropometric measurement (interaction P = .91)			
6-8 mo.	-0.32(1.24)	-0.27(1.30)	0.05 (-0.26, 0.36)
12-14 mo.	-1.48(1.08)	-1.33(1.19)	0.1 (-0.21, 0.41)
WAZ at first MDA exposure (interaction P = .17)			
>= -2 SD	-0.85(1.27)	-0.63(1.42)	0.21 (-0.15, 0.58)
< -2 SD	-1.46(1.35)	-2.00(1.16)	-0.33 (-1.04, 0.38)
SMC given 3 months before (interaction P = .69)			
Yes	-1.33(0.82)	-0.92(2.39)	-0.21 (-1.94, 1.53)
No	-0.83(1.33)	-0.62(1.38)	0.23 (-0.06, 0.52)
Asset index (interaction P = .47)			
Above median	-0.88(1.34)	-0.74(1.45)	0.24 (-0.1, 0.58)
At or below median	-0.94(1.27)	-0.87(1.36)	0.03 (-0.31, 0.37)
WASH index (interaction P = .15)			
Above median	-0.90(1.33)	-0.98(1.36)	-0.03 (-0.37, 0.3)
At or below median	-0.92(1.28)	-0.65(1.44)	0.31 (-0.04, 0.65)

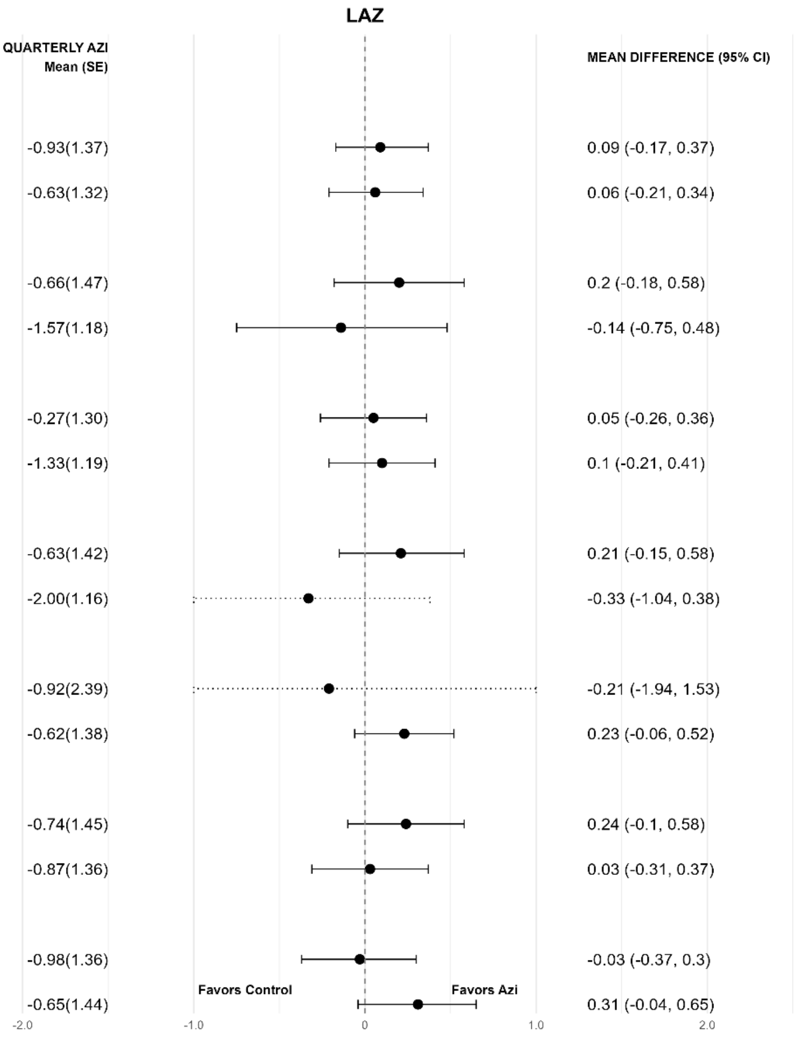

© 2026 Adubra L et al. *JAMA Network Open.*

eFigure 3c. Mean Differences in Weight-for-Length z Scores (WLZ) Between Control and Quarterly Azithromycin Groups by Child and Household Characteristics[a]

[a]Data were collected at mass drug administration (MDA) rounds 6–8 and the close-out visit (15, 18, 21, and 24 months after village enrollment). Infants received azithromycin or placebo between ages 1 and 11 months. Displayed values are observed (raw) group means ± SD. Estimates (mean difference, 95% CI) are shown for Azithromycin quarterly vs Placebo. P values are from global likelihood ratio tests of subgroup × intervention interaction including all study arms (Control, Twice-yearly, Quarterly). Confidence intervals extending beyond the axis range are truncated in the figure and shown with dotted lines.

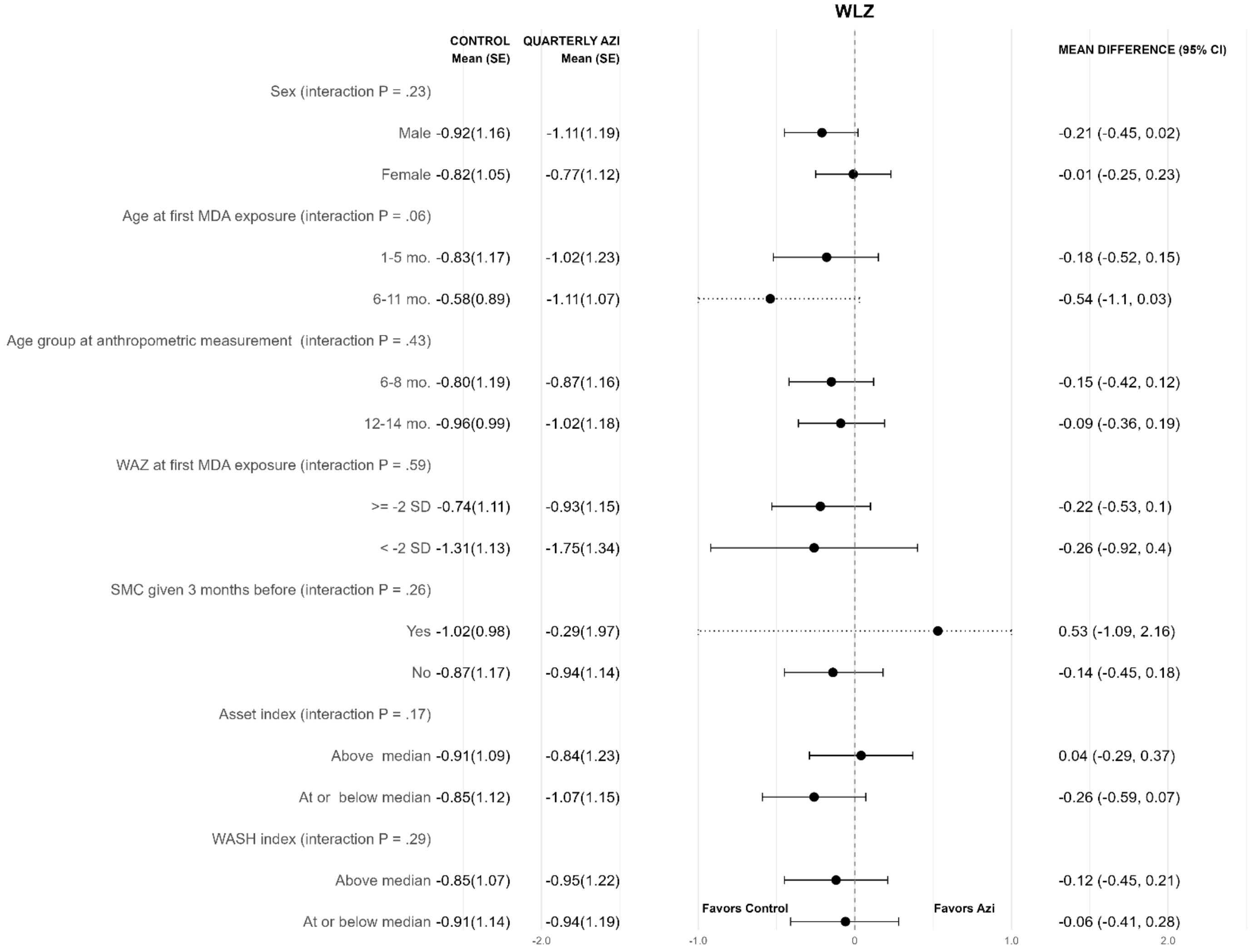

© 2026 Adubra L et al. *JAMA Network Open.*

eFigure 3d. Mean Differences in Mid-Upper-Arm Circumference z Scores (MUACZ) Between Control and Quarterly Azithromycin Groups by Child and Household Characteristics[a]

[a]Data were collected at mass drug administration (MDA) rounds 6–8 and the close-out visit (15, 18, 21, and 24 months after village enrollment). Infants received azithromycin or placebo between ages 1 and 11 months. Displayed values are observed (raw) group means ± SD. Estimates (mean difference, 95% CI) are shown for Azithromycin quarterly vs Placebo. P values are from global likelihood ratio tests of subgroup × intervention interaction including all study arms (Control, Twice-yearly, Quarterly). Confidence intervals extending beyond the axis range are truncated in the figure and shown with dotted lines.

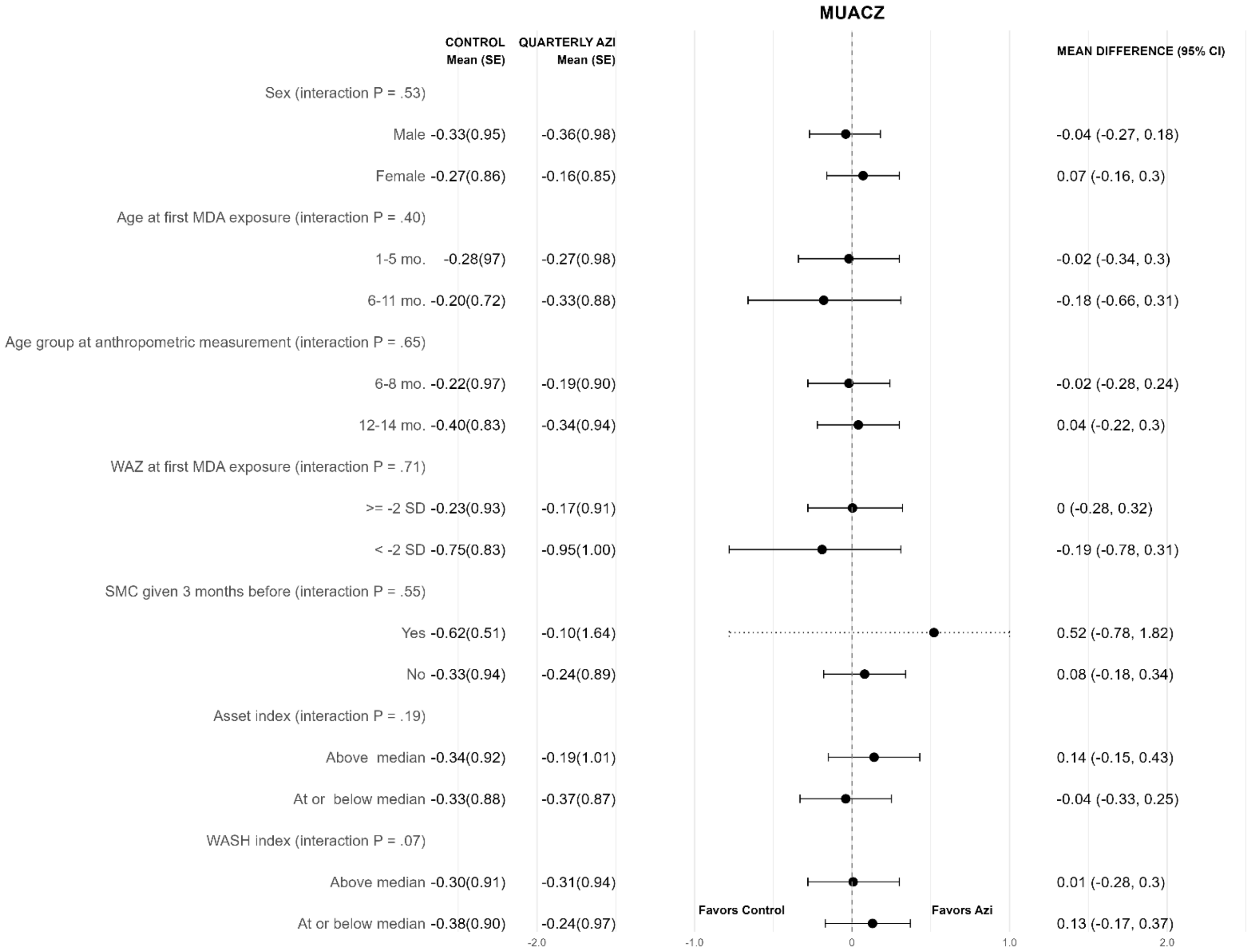

© 2026 Adubra L et al. *JAMA Network Open.*

eFigure 4a. Mean Differences in Weight-for-Age z Scores (WAZ) Between Control and Quarterly Azithromycin Groups by Ecological Characteristics[a]

[a]Data were collected at mass drug administration (MDA) rounds 6–8 and the close-out visit (15, 18, 21, and 24 months after village enrollment). Infants received azithromycin or placebo between ages 1 and 11 months. Displayed values are observed (raw) group means ± SD. Estimates (mean difference, 95% CI) are shown for Azithromycin quarterly vs Placebo. P values are from global likelihood ratio tests of subgroup × intervention interaction including all study arms (Control, Twice-yearly, Quarterly).

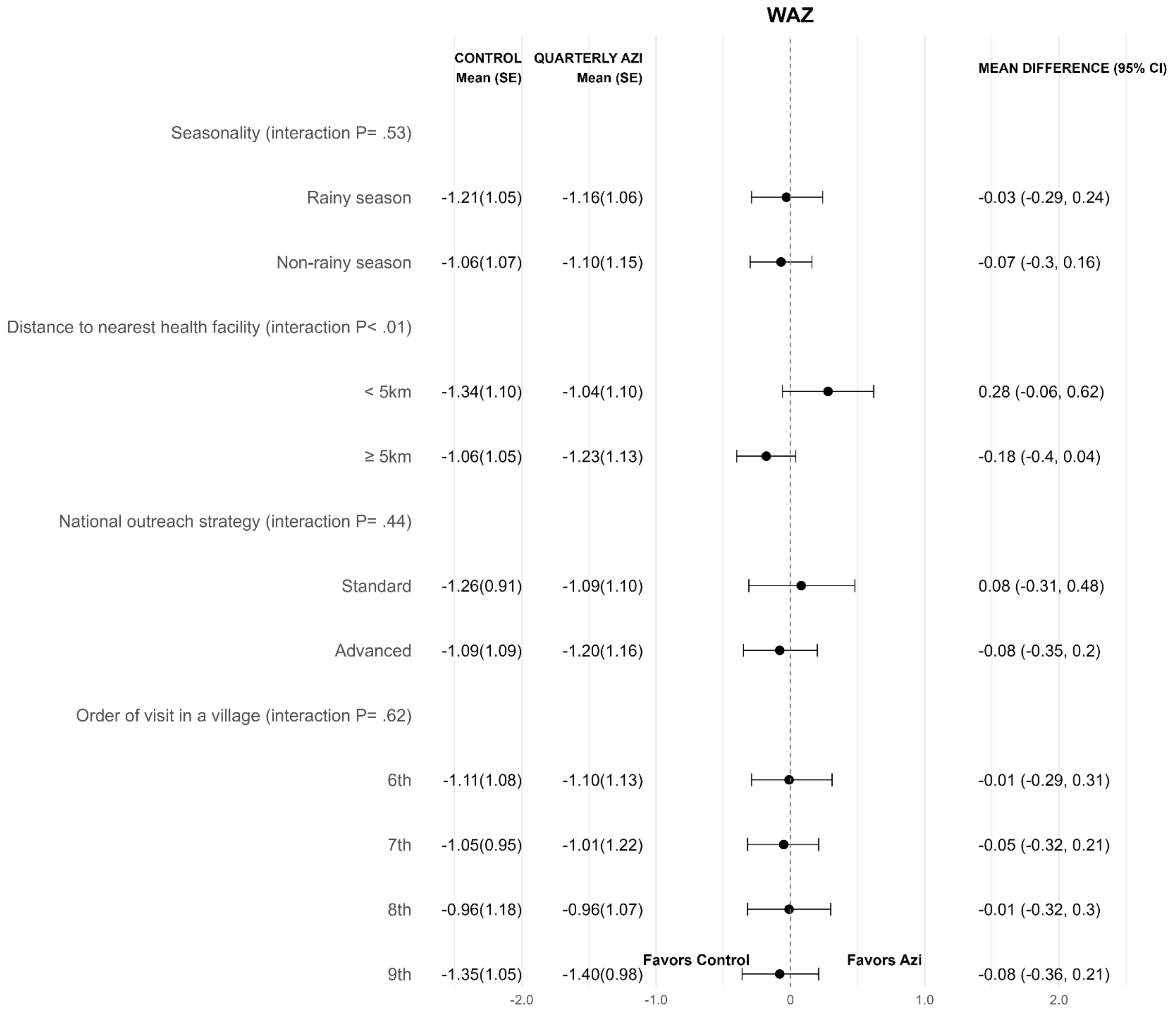

© 2026 Adubra L et al. *JAMA Network Open.*

eFigure 4b. Mean Differences in Length-for-Age z Scores (LAZ) Between Control and Quarterly Azithromycin Groups by Ecological Characteristics[a]

[a]Data were collected at mass drug administration (MDA) rounds 6–8 and the close-out visit (15, 18, 21, and 24 months after village enrollment). Infants received azithromycin or placebo between ages 1 and 11 months. Displayed values are observed (raw) group means ± SD. Estimates (mean difference, 95% CI) are shown for Azithromycin quarterly vs Placebo. P values are from global likelihood ratio tests of subgroup × intervention interaction including all study arms (Control, Twice-yearly, Quarterly).

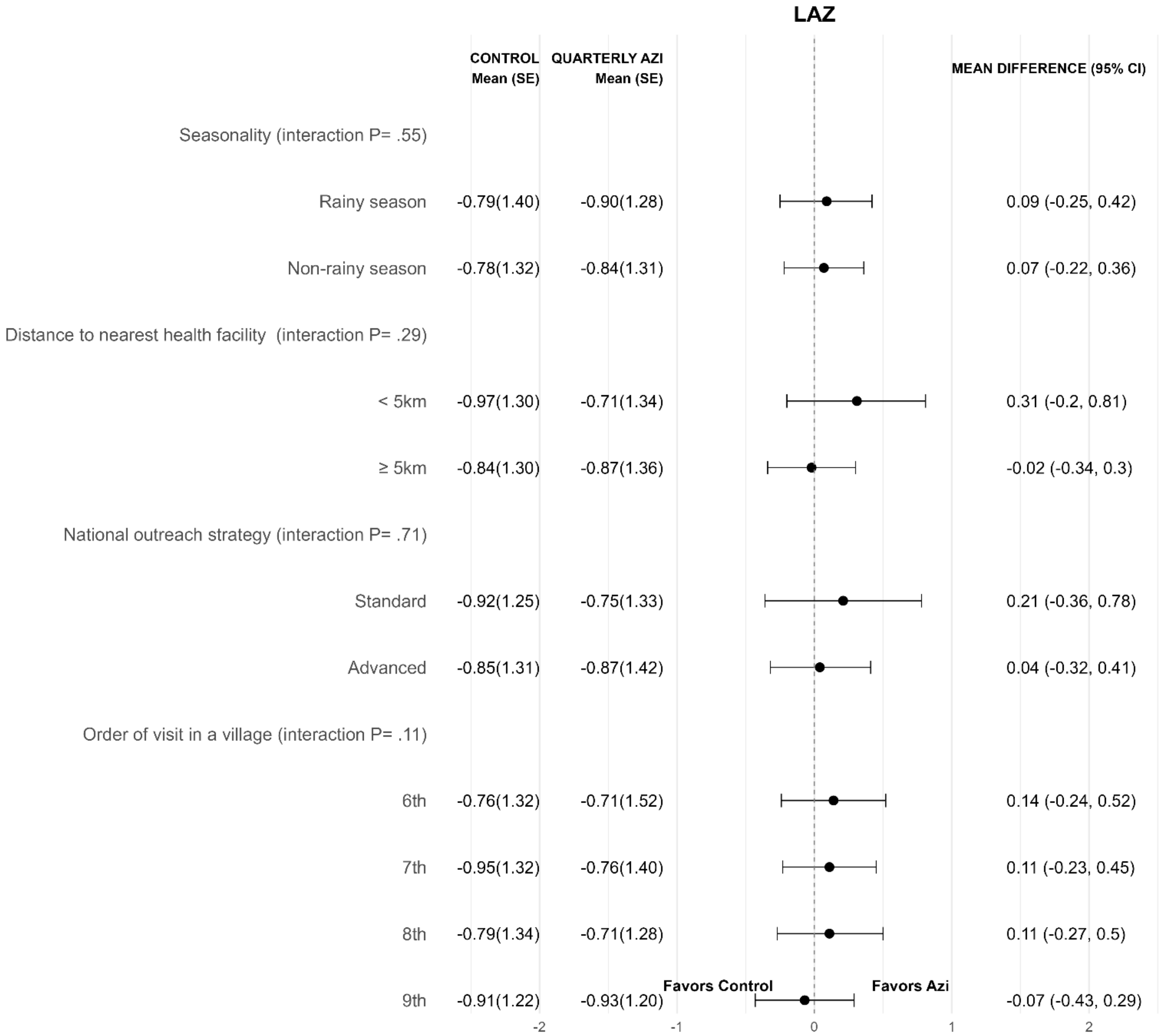

© 2026 Adubra L et al. *JAMA Network Open.*

eFigure 4c. Mean Differences in Weight-for-Length z Scores (WLZ) Between Control and Quarterly Azithromycin Groups by Ecological Characteristics[a]

[a]Data were collected at mass drug administration (MDA) rounds 6–8 and the close-out visit (15, 18, 21, and 24 months after village enrollment). Infants received azithromycin or placebo between ages 1 and 11 months. Displayed values are observed (raw) group means ± SD. Estimates (mean difference, 95% CI) are shown for Azithromycin quarterly vs Placebo. P values are from global likelihood ratio tests of subgroup × intervention interaction including all study arms (Control, Twice-yearly, Quarterly).

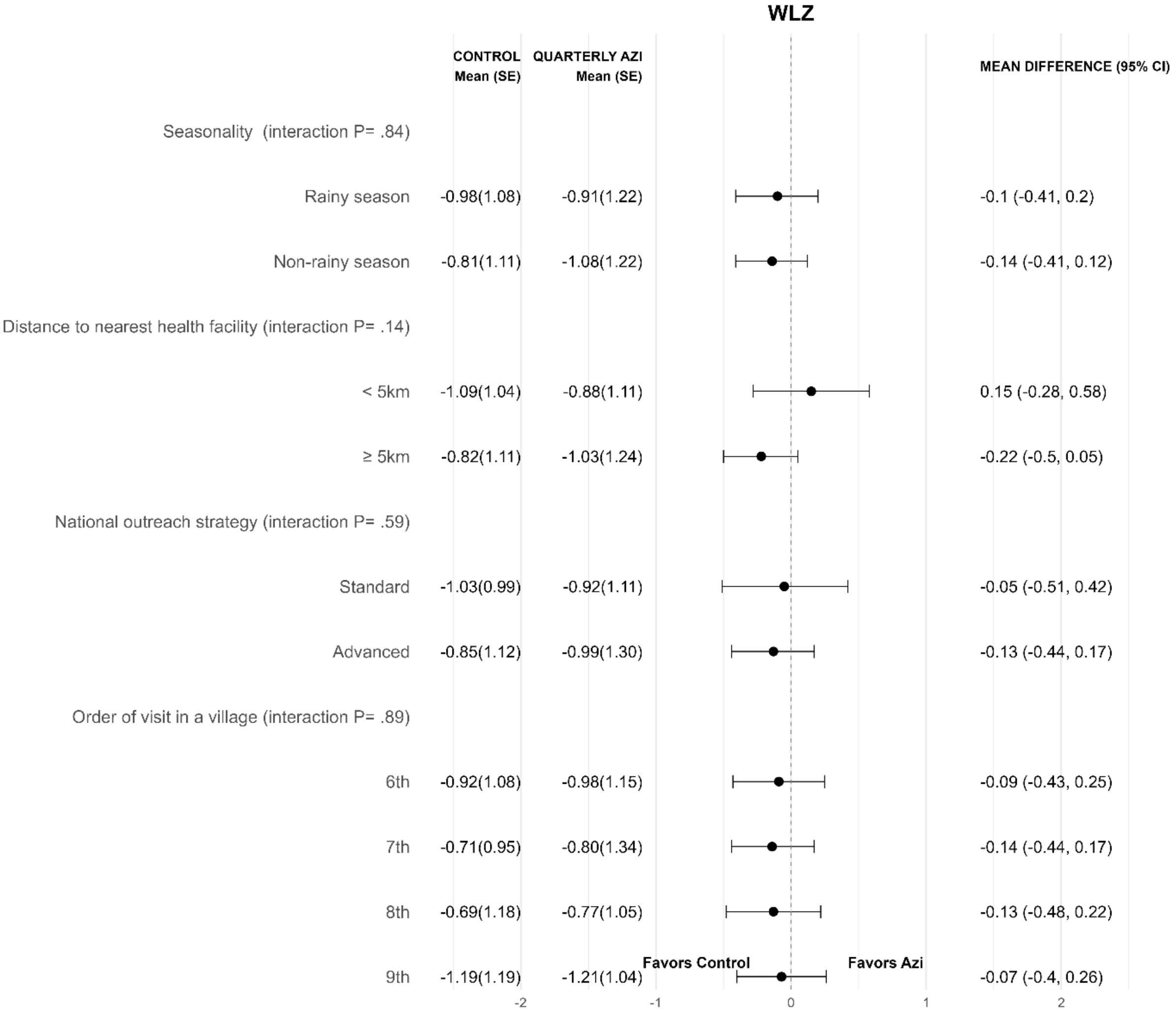

© 2026 Adubra L et al. *JAMA Network Open.*

eFigure 4d. Mean Differences in Mid-Upper-Arm Circumference z Scores (MUACZ) Between Control and Quarterly Azithromycin Groups by Ecological Characteristics[a]

[a]Data were collected at mass drug administration (MDA) rounds 6–8 and the close-out visit (15, 18, 21, and 24 months after village enrollment). Infants received azithromycin or placebo between ages 1 and 11 months. Displayed values are observed (raw) group means ± SD. Estimates (mean difference, 95% CI) are shown for Azithromycin quarterly vs Placebo. P values are from global likelihood ratio tests of subgroup × intervention interaction including all study arms (Control, Twice-yearly, Quarterly).

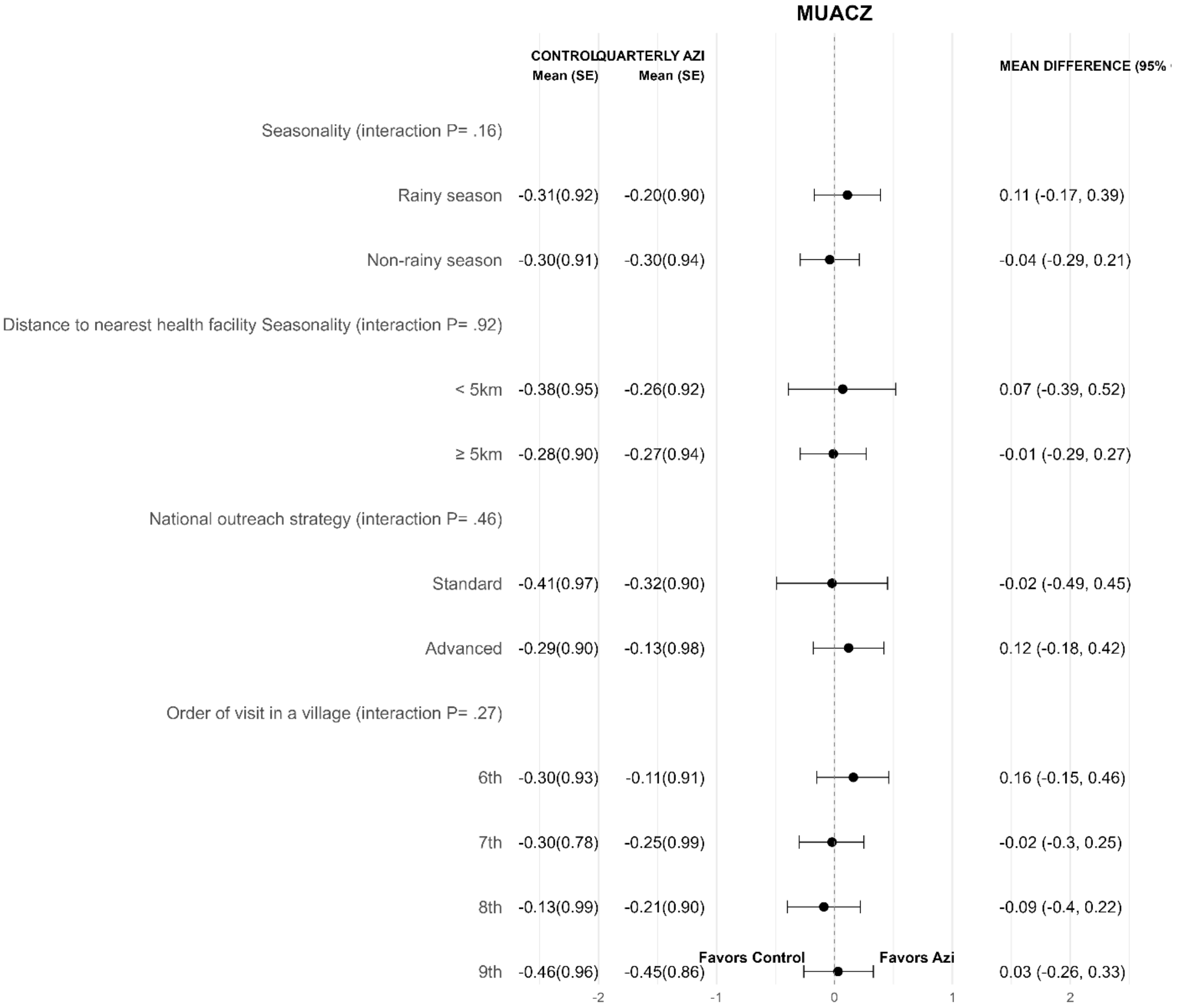

© 2026 Adubra L et al. *JAMA Network Open.*
