# Supplementary material for: Infant Growth After Mass Administration of Azithromycin: Secondary Outcomes of a Cluster Randomized Clinical Trial
Source: JAMA Netw Open. 2026 Jun 10;9(6):e2617425. doi: 10.1001/jamanetworkopen.2026.17425 (PMC13254738; doi:10.1001/jamanetworkopen.2026.17425)
Supplement: Supplement 3. — Data Sharing Statement [file jamanetwopen-e2617425-s003.pdf]

Data Sharing Statement

Adubra. Infant Growth After Mass Administration of Azithromycin. *JAMA Netw Open*. Published June 10, 2026. doi:10.1001/jamanetworkopen.2026.17425

Data
Additional Information: ClinicalTrials.gov ID NCT04424511
https://clinicaltrials.gov/study/NCT04424511?cond=lakana%20trial&rank=1
Data available: Yes
Data types: Deidentified participant data, Other (please specify)
Additional Information: Analytical Code, Protocol full text (original and latest versions)
How to access data: LAKANA Growth data + data dictionary:
https://doi.org/10.5281/zenodo.17404687
When available: beginning date: 10-21-2025

Supporting Documents
Document types: Other (please specify)
Additional Information: Protocol full text (original and latest versions)
How to access documents: LAKANA Growth Analytical Code + LAKANA Protocol:
https://doi.org/10.5281/zenodo.17414478
When available: beginning date: 10-22-2025

Additional Information
Who can access the data: LAKANA Growth Substudy data have been deposited in Zenodo accessible via the link provided. Because open data sharing was not included in the study information sheet at the time of data collection, access to the dataset is restricted. Access requests should be submitted to the LAKANA Trial Principal Investigators at lakana.psg@lists.tuni.fi
Types of analyses: Data will be made available to users who submit a written request outlining their proposed use and providing a justification and data use plan.
Mechanisms of data availability: Data will be made available to users who submit a written request outlining their proposed use and providing a justification and data use plan.
